# Metal–Organic Framework and Covalent–Organic Framework‐Based Aerogels: Synthesis, Functionality, and Applications

**DOI:** 10.1002/advs.202409290

**Published:** 2024-10-28

**Authors:** Gaofeng Shao, Xiaogu Huang, Xiaodong Shen, Changxia Li, Arne Thomas

**Affiliations:** ^1^ School of Chemistry and Materials Science Jiangsu Key Laboratory of New Energy Devices and Interface Science Nanjing University of Information Science and Technology Nanjing 210044 China; ^2^ College of Materials Science and Engineering Nanjing Tech University Nanjing 211816 China; ^3^ School of Chemistry and Molecular Engineering Nanjing Tech University Nanjing 211816 China; ^4^ Department of Chemistry School of Science Westlake University 600 Dunyu Road Hangzhou Zhejiang 310024 China; ^5^ Institute for Chemistry Division of Functional Materials Technische Universität Berlin 10623 Berlin Germany

**Keywords:** aerogel, covalent–organic framework, hybrids, metal–organic framework, porous materials

## Abstract

Metal–organic frameworks (MOFs) and covalent–organic frameworks (COFs)‐based aerogels are garnering significant attention owing to their unique chemical and structural properties. These materials harmoniously combine the advantages of MOFs and COFs—such as high surface area, customizable porosity, and varied chemical functionality—with the lightweight and structured porosity characteristic of aerogels. This combination opens up new avenues for advanced applications in fields where material efficiency and enhanced functionality are critical. This review provides a comparative overview of the synthetic strategies utilized to produce pristine MOF/COF aerogels as well as MOF/COF‐based hybrid aerogels, which are functionalized with molecular precursors and nanoscale materials. The versatility of these aerogels positions them as promising candidates for addressing complex challenges in environmental remediation, energy storage and conversion, sustainable water‐energy technologies, and chemical separations. Furthermore, this study discusses the current challenges and future prospects related to the synthesis techniques and applications of MOF/COF aerogels.

## Graphical abstract

This review paper focuses on metal–organic frameworks (MOFs) and covalent–organic frameworks (COFs)‐based aerogels, which provide an extensive description of the synthetic strategies and structural features of MOF/COF‐based aerogels, along with their cutting‐edge applications in the realm of environmental remediation, gas storage and separation, sustainable energy‐water technology, energy storage and conversion.

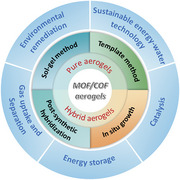

## 1 Introduction

Aerogels have gained significant interest over the past few decades due to their exceptional properties, which include high porosity, adjustable densities, extensive surface areas, and voluminous pore spaces. Aerogels are prepared usually from molecular precursors via sol‐gel routes to obtain a gel, followed by supercritical drying or freeze‐drying to remove the liquid from the wet gels without collapsing their interconnected porous network.^[^ ^1^, ^2^, ^3^ ^]^ Aerogel pore sizes are highly versatile, ranging from micropores to mesopores, and extending up to macropores.^[^ ^4^, ^5^ ^]^ Since the invention of the first aerogel by Kistler in 1931,^[^ ^6^ ^]^ a plethora of research has been conducted, resulting in the development of various types of aerogels. These include silica,^[^ ^7^, ^8^, ^9^ ^]^ metal oxide,^[^ ^10^, ^11^, ^12^ ^]^ metal,^[^ ^13^, ^14^, ^15^ ^]^ non‐oxide ceramics (borides,^[^ ^16^ ^]^ nitrides,^[^ ^17^, ^18^, ^19^, ^20^ ^]^ carbides,^[^ ^21^, ^22^, ^23^ ^]^ and MXene^[^ ^24^ ^]^), semiconductor,^[^ ^25^, ^26^ ^]^ biomass,^[^ ^27^, ^28^, ^29^, ^30^, ^31^ ^]^ polymer,^[^ ^32^, ^33^ ^]^ and carbon (graphene,^[^ ^34^, ^35^ ^]^ carbon nanotube,^[^ ^36^, ^37^ ^]^ carbon nanofiber^[^ ^38^, ^39^ ^]^) aerogels. These aerogels have found extensive applications in various fields encompassing thermal insulation,^[^ ^40^, ^41^, ^42^, ^43^ ^]^ radiative cooling,^[^ ^44^ ^]^ environmental remediation,^[^ ^45^, ^46^ ^]^ catalysis,^[^ ^47^, ^48^ ^]^ and energy storage.^[^ ^49^, ^50^ ^]^ Industrial utilization of silica aerogels, in particular, spans multiple sectors, including oil and gas, aerospace, and construction, where they shine as thermal insulators and lightweight structural elements.^[^ ^8^ ^]^ Beyond silica, aerogels made of polymers, carbon, and graphene are demonstrating potential for industrial use in fields such as energy storage, sensory technology, and electronics. However, several types of aerogels, notably those comprised of metal oxides and biopolymers, are predominantly explored within research settings at present. Investigations are ongoing to unveil their potential for specialized commercial applications in niche markets.^[^ ^51^ ^]^

Metal–organic frameworks (MOFs) and covalent–organic frameworks (COFs), which are two distinct classes of crystalline porous materials,^[^ ^52^, ^53^, ^54^, ^55^ ^]^ have emerged as appealing alternatives in the area of advanced adsorption, separation and purification,^[^ ^56^, ^57^, ^58^ ^]^ catalysis,^[^ ^59^, ^60^, ^61^, ^62^, ^63^, ^64^, ^65^ ^]^ energy storage,^[^ ^66^, ^67^ ^]^ sustainable energy‐water technology,^[^ ^68^, ^69^, ^70^ ^]^ besides many other promising applications, because of their well‐defined pore structures and adjustable surface properties.^[^ ^71^, ^72^ ^]^ MOFs and COFs are constructed from inorganic metal ion/metal cluster or organic building units and organic linkers through coordination or covalent bonds, respectively.^[^ ^73^, ^74^ ^]^ The large variability in structure and size of building blocks, can yield many different network topologies and therefore MOFs/COFs with versatile structures and properties can be created.^[^ ^75^, ^76^, ^77^, ^78^ ^]^ However, after synthesis, MOFs/COFs are usually obtained in the form of fine powders, which limits their processability and large‐scale practical applications. With the development of advanced fabrication techniques to create three‐dimensional (3D) aerogels and the possibility to adapt these for MOF/COF synthesis, MOF/COF aerogels and their hybrid aerogels have been emerged in the recent decade.

A number of reviews on hierarchical MOF‐based aerogels have recently been published.^[^ ^79^, ^80^, ^81^ ^]^ With the development of COF‐based aerogels in very recent years,^[^ ^82^, ^83^, ^84^, ^85^, ^86^ ^]^ it seems sensible to provide a comprehensive overview on both types of aerogels, pointing out the similarities and differences between MOF and COF aerogels. In this review, we therefore systematically summarize the recent progress of MOF‐ and COF‐based aerogels regarding the synthesis strategies and their cutting‐edge applications.

**Figure 1:**
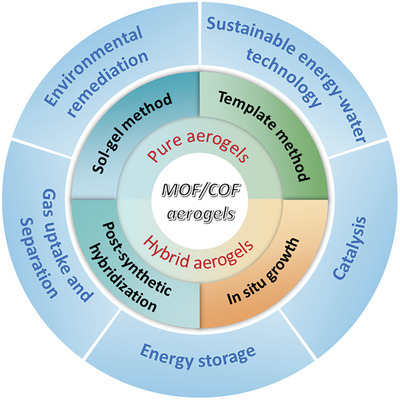
Schematic representation of the synthetic strategies and various applications of MOF/COF‐based aerogels.

As outlined in **Figure** **1**, the review starts describing the synthetic strategies for pristine MOF/COF aerogels, mainly including sol‐gel and templating methods. Subsequently, the recent progress in MOF/COF‐based hybrid aerogels is summarized, in which MOF/COF nanocrystals are composited with molecular precursors and nanoscale materials such as zero‐dimensional (0D) nanoparticles, one‐dimensional (1D) nanofibers/nanotubes, or two‐dimensional (2D) nanosheets. Third, emerging functional applications are presented, including environmental remediation, gas uptake and separation, sustainable energy‐water technology, energy storage and conversion, and electromagnetic wave absorbing and shielding. Finally, the challenges and prospects of MOF/COF aerogels are briefly discussed.

## 2 Synthetic Strategies of MOF/COF Aerogels

### 2.1 Sol‐Gel Method

**Figure 2:**
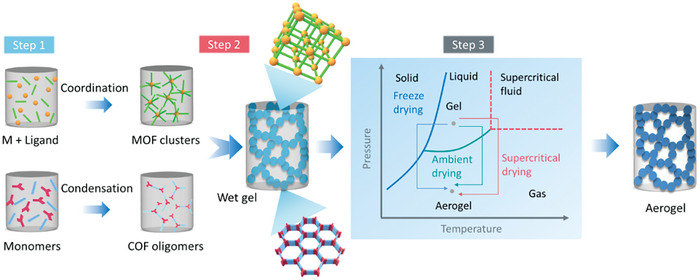
Schematic overview of the various steps involved in the synthesis of pristine MOF/COF aerogels by sol‐gel method.

**Figure 3:**
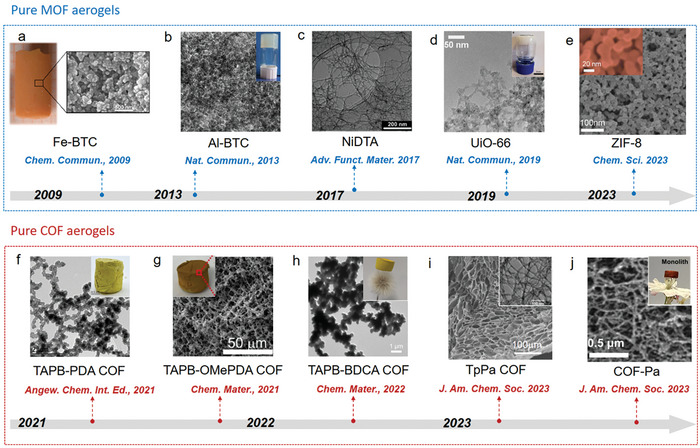
Timeline of typical MOF/COF aerogels. a) Fe‐BTC aerogel. Reproduced with permission.^[^ ^88^ ^]^ Copyright 2009, Royal Society of Chemistry. b) Al‐BTC aerogel.^[^ ^87^ ^]^ Reproduced under the terms of the Creative Commons CC‐BY license. Copyright 2013, Springer Nature. c) NiDTA aerogel. Reproduced with permission.^[^ ^90^ ^]^ Copyright 2017, Wiley‐VCH. d) UiO‐66 xerogel. Reproduced under the terms of the Creative Commons CC‐BY license.^[^ ^91^ ^]^ Copyright 2019, Springer Nature. e) ZIF‐8 aerogel. Reproduced with permission.^[^ ^89^ ^]^ Copyright 2023, Royal Society of Chemistry. f) TAPB‐PDA COF aerogel. Reproduced with permission.^[^ ^82^ ^]^ Copyright 2021, Wiley‐VCH. g) TAPB‐OMePDA COF aerogel. Reproduced with permission.^[^ ^83^ ^]^ Copyright 2021, American Chemical Society. h) TAPB‐BDCA COF aerogel. Reproduced with permission.^[^ ^84^ ^]^ Copyright 2022, American Chemical Society. i) TpPa COF aerogel. Reproduced with permission.^[^ ^85^ ^]^ Copyright 2023, American Chemical Society. j) COF‐Pa aerogel. Reproduced with permission.^[^ ^86^ ^]^ Copyright 2023, American Chemical Society.

The sol‐gel method is probably the most common strategy for the preparation of aerogels, and can be well applied to synthesize MOF or COF wet gels with 3D interconnected network structures. Starting from MOF/COF precursor solutions, gels are formed followed by solvent exchange and drying steps to obtain the corresponding aerogels.^[^ ^82^, ^83^, ^84^, ^85^, ^87^, ^88^, ^89^ ^]^ As shown in **Figure** **2**, in the first step, primary MOF clusters or COF oligomers are initially formed by metal–ligand coordination or condensation reaction of COF monomers. In the second step, gelation occurs, involving the formation of highly porous, interconnected networks of nanoparticles. This happens through the mismatched growth or cross‐linking of the MOF clusters, which is triggered by changes in temperature or chemical conditions. In the case of COF aerogels, the gelation can be triggered by acid catalysis in a suitable solvent, resulting in the formation of COF wet gels. The final step involves drying of the MOF and COF wet gels to preserve their structural integrity. The most common drying technique in sol‐gel derived wet gels is supercritical CO_2_ drying, which can yield hierarchical porous structures. Often, it is essential to exchange the pore liquid of these gels with ethanol due to its better compatibility with the supercritical point drying procedure. Another method is to first produce a dense wet gel through centrifugation, followed by ambient pressure drying, resulting in a high‐density aerogel, also called xerogel. At present, there is abundant literature on the factors influencing the formation of MOF aerogels and corresponding drying methods, while the research on COF aerogels has just emerged in recent years (**Figure** **3**).

#### 2.1.1 The Formation of MOF Aerogels

Since the pioneering work in 2009 by Kaskel et al. on Fe‐BTC (BTC = 1,3,5‐benzene tricarboxylate) MOF aerogels, which were prepared using a sol‐gel method and dried with supercritical CO_2_ (Figure 3a),^[^ ^88^ ^]^ various kinds of MOF aerogels (MOFAs) including monometallic (Fe,^[^ ^92^ ^]^ Cr,^[^ ^92^ ^]^ Al,^[^ ^87^, ^93^ ^]^ Ga,^[^ ^87^ ^]^ In,^[^ ^87^ ^]^ Rh,^[^ ^94^ ^]^ and Zr^[^ ^95^ ^]^) or binary (Fe‐Al^[^ ^96^ ^]^) MOFAs coordinated with rigid bridging carboxylate ligands have been synthesized (**Table** **1**). Very recently, Yeung's group proposed a general gel transformation strategy for the fabrication of various MOF aerogels based on Al, Co, Cu, Eu, Fe, Ni, and Zn gels, featuring low density, high mesoporosity, permeability, and improved strength.^[^ ^89^ ^]^ These materials simultaneously process well‐ordered intraparticle micropores and aerogel‐specific interparticle mesopores.

**Table 1:** Synthesis conditions, physical properties, and suggested applications of MOF aerogels prepared by the sol‐gel method.

MOF aerogels	Metal sources	Ligands	Solvent	T	Drying method	ρ_b_ [g cm^−3^]	V_tot_ [cm^3^ g^−1^]	S_BET_ [m^2^ g^−1^]	Applications	Refs.
Fe‐BTC	Fe(NO_3_)_3_	H_3_BTC	EtOH	RT	SD	0.0145–0.1105	5.62	1618	–	2009^[^ ^88^ ^]^
					APD	–	0.71	1200	–	
Fe‐BTC	Fe(NO_3_)_3_·9H_2_O	H_3_BTC	EtOH	RT	SD		0.67	1016	CO_2_ uptake	2010^[^ ^102^ ^]^
Fe‐BTC		H_3_BTC				–	1.358	1090	–	2012^[^ ^92^ ^]^
Fe‐BuDC		H_2_BuDC				–	1.991	424	–	
Fe‐BDC		H_2_BDC	EtOH‐DMF	80 °C		–	3.146	1454	–	
Cr‐BTC	Cr (NO_3_)_3_·9H_2_O	H_3_BTC	EtOH			–	0.437	570	Dye adsorption	
Cr‐BDC		H_2_BDC	EtOH‐DMF			–	1.527	737	–	
Cr‐NDC		H_2_NDC	EtOH			–	1.489	491	–	
Cr‐ADC		H_2_ADC				–	0.912	314	–	
Cr‐FDC		H_2_FDC				–	0.401	112	–	
Cr‐BuDC		H_2_BDC				–	0.085	54	–	
Cr‐BTB		H_3_BTB	EtOH‐DMF			–	0.067	29	–	
Al‐BDC	Al(NO_3_)_3_·9H_2_O	H_2_BDC	EtOH			0.299	6.27	1560	Dye adsorption	2013^[^ ^87^ ^]^
Al‐BTC		H_3_BTC					6.26	1638	CO_2_ uptake	
Ga‐BTC	Ga(NO_3_)_3_ nH_2_O	H_3_BTC		40 °C	–	–	–	–	–	
In‐BDC	In(NO_3_)_3_ nH_2_O	H_2_BDC	EtOH‐DMF	80 °C	–	–	–	–	–	
Al‐BTC	Al(NO_3_)_3_·9H_2_O	H_3_BTC	EtOH	120 °C	SD	–	4.5	1795	Microcystin‐LR removal	2013^[^ ^93^ ^]^
					APD	–	1.31	1761		
Al‐ACAC	Al(NO_3_)_3_·9H_2_O	acetylacetonate	DMF‐EtOH	85 °C	SD	–	0.78	1040	Gas and vapor uptake	2014^[^ ^97^ ^]^
Cr‐ACAC	Cr (NO_3_)_3_·9H_2_O		DMF‐DMF			–	2.65	717		
Al‐Mg‐ACAC	MgCl_2_·6H_2_O		DMF‐EtOH			–	1.32	1026		
Al‐Ca‐ACAC	CaCl_2_		DMF‐EtOH			–	1.38	1138		
Fe‐Al‐BTC	Fe(NO_3_)_3_·9H_2_O, Al(NO_3_)_3_·9H_2_O	H_3_BTC	EtOH	120 °C	SD		9.737	1861	Dye adsorption	2015^[^ ^96^ ^]^
					APD		0.88	1289		
ZIF‐8	Zn(NO_3_)_2_·6H_2_O	2MIM	EtOH	RT	VD	1.05	0.546	1423	Gas uptake	2015^[^ ^99^ ^]^
Rh‐ BTCTB	Rh_2_(OAc)_4_	H_3_BTCTB	DMF/H_2_O	85 °C	SD	–	0.79	230	Dye adsorption	2016^[^ ^94^ ^]^
Rh‐ BTCTB			DMF/MeOH			–	1.12	405		
UiO‐66	ZrOCl_2_·8H_2_O	H_2_BDC	DMF	100 °C	APD	0.386	2.09	1459	–	2017^[^ ^95^ ^]^
					SD	–	1.66	1255	–	
NiDTA	Ni(OAc)_2_	H_2_DTA	DMF/DMA	15 °C	SD	0.03	1.406	427	‐	2017^[^ ^90^ ^]^
PdDTA	Pd(OAc)_2_					0.49	0.441	189		
CuDTA	Cu(OAc)_2_					0.05	2.243	211		
NiPdDTA	Ni(OAc)_2_, Pd(OAc)_2_					0.08	3.917	307		
NiCuDTA	Ni(OAc)_2_, Cu(OAc)_2_					0.04	2.508	372		
PdCuDTA	Pd(OAc)_2_, Cu(OAc)_2_					0.11	1.742	248		
Cu‐BTC	Cu(NO_3_)_2_ 2.5H_2_O	H_3_BTC	EtOH	RT	APD	1.06	0.52	1193	CH_4_ uptake	2018
UiO‐66	ZrOCl_2_∙8H_2_O	H_3_BTC	DMF	90	SD	–	–	390		2018^[^ ^98^ ^]^
UiO‐66	ZrOCl_2_∙8H_2_O	H_2_BDC		100 °C	APD	0.43	1.62	1177	CO_2_ and CH_4_ uptake	2019^[^ ^91^ ^]^
UiO‐66‐NH_2_	ZrOCl_2_∙8H_2_O	H_2_BDC‐NH_2_	DMF	80 °C	VD	–	0.51	863	–	2019^[^ ^103^ ^]^
CAU‐3	Al(NO_3_)_3_·9H_2_O	H_2_BDC	MeOH	120 °C	APD	–	–	2050	Vapor uptake	2020^[^ ^104^ ^]^
ZIF‐8	Zn(NO_3_)_2_·6H_2_O	2MIM	MeOH	RT		1.17	1.117	1597	Vapor uptake	2021^[^ ^100^ ^]^
ZIF‐67	Co(NO_3_)_2_·6H_2_O					1.11	1.647	1731		
ZIF‐8	Zn(NO_3_)_2_·6H_2_O	2MIM	MeOH		SD	0.423	1.35	1312	–	2023^[^ ^89^ ^]^
MOF‐5	Zn(NO_3_)_2_·6H_2_O	H_2_BDC	DMF			0.476	1.47	1456		

Note: *T* = gelation temperature, SD = supercritical drying, APD = ambient pressure drying, VD = vacuum drying, *S*_BET_ = BET specific area, V_tot_ = total pore volume, ρ_b_ = bulk density

The critical step of the MOFA synthesis lies in the sol‐gel process, which is responsible for the development of 3D networks and significantly influences the properties of the final material. During the sol‐gel process, the metal ion source,^[^ ^90^, ^92^, ^97^ ^]^ solvent,^[^ ^87^, ^90^, ^92^ ^]^ and temperature^[^ ^87^, ^92^, ^93^ ^]^ have a key role influencing the initial nucleation and triggering the gelation of MOF instead of their precipitation or crystallization. Other factors, such as the metal‐to‐organic ratio^[^ ^87^, ^92^ ^]^ and precursor concentration,^[^ ^90^, ^94^, ^98^ ^]^ typically affect the gelation time and porosity of the resulting aerogels. Ideally, the structure of the wet gel is directly transferred to the aerogel. Therefore, a thorough understanding of the gelation process is crucial for successful aerogel synthesis.

*Temperature*: In‐depth studies of the influence of temperature on gelation process were carried out by Zhang and Su et al.^[^ ^87^, ^92^ ^]^ Taking Al‐BDC (BDC = 1,4‐benzenedicarboxylate) MOF gel as a model system (Figure 3b),^[^ ^87^ ^]^ the formation of the MOF undergoes two stages: 1) metal ions and ligands assemble into primary MOF clusters driven by metal–ligand coordination, then polymerize to initiate the nucleation process. With the decrease of the concentration of metal ions and ligands, the nucleation of new particles is decelerated. 2) The given temperature determines if coordination whether continues or is perturbed. At higher temperature, MOF particles can form and finally crystallize. However, under milder heating conditions (80 °C in this instance), the reversibility of the coordination bonding decrease, leading to mismatched growth or cross‐linking. These conditions provide the necessary prerequisites for the gelation process at this stage. Additionally, the team prepared Cr^3+^/Fe^3+^ gels through the straightforward method of mixing a metal salt solution and a variety of bridging carboxylic acids.^[^ ^92^ ^]^ Most of the ligands can form gels with Fe^3+^ at room temperature. However, the formation of Fe‐BDC gel, similar to all of the Cr^3+^‐containing gels, requires elevated temperatures (above 80 °C), as they only form when the temperature reaches this threshold.

*Solvent*: The influence of solvents has also been investigated. Many metal–organic gels can be formed in alcohols. However, if the system is strictly anhydrous (e.g., mixing anhydrous FeCl_3_ and H_3_BTC in absolute ethanol), no gelation was observed. Therefore, small amounts of water may facilitate the gelation process, as water can stabilize the gel network through extensive hydrogen bonding or contribute to formation of oxo‐bridged clusters.^[^ ^92^ ^]^ Additionally, dithiooxalate (DTA) has been utilized as ligand for the fabrication of electrical conductive MOFAs. It is capable of bridging transition metal ions in a bis‐bidentate coordination mode (Figure 3c).^[^ ^90^ ^]^ A series of organic solvents, such as N,N‐dimethylformamide (DMF), dimethylsulfoxide (DMSO), N, N‐dimethylacetamide (DEA), N,N‐dimethylacetamide (DMA), methanol (MeOH), ethanol (EtOH), dichloromethane (CH_2_Cl_2_), chloroform (CHCl_3_), acetonitrile (CH_3_CN), formamide, and acetone, were used to investigate the formation of Ni‐DTA. Among these, only solvents with higher coordinative ability and steric hindrance, specifically DMF, DMSO, and DEA which process hydrogen bonding acceptor capabilities, were found to facilitate gelation.

**Figure 4:**
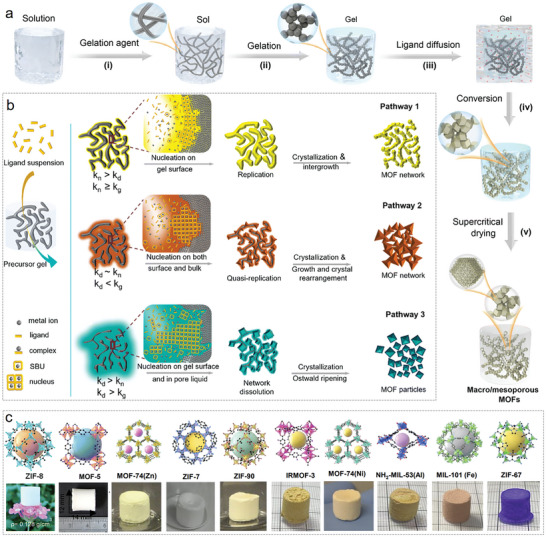
a) Synthesis route of MOF aerogels. b) Three different sol‐gel transformation pathways. c) Crystal structures and corresponding photographs of MOF aerogels. Reproduced with permission.^[^ ^89^ ^]^ Copyright 2023, Royal Society of Chemistry.

*Gelation Agent*: A latest progress in the fabrication of MOF aerogels was achieved by a gel transformation strategy.^[^ ^89^ ^]^ Specifically, the metal clusters were firstly assembled into gel networks using epoxide as the gelation agent. Subsequently, organic ligands were introduced to react with metal precursor and transform the gel network into crystallized MOF gels (**Figure** **4**a). By manipulating the dissolution, nucleation, and crystallization kinetics during the transformation process (Figure 4b), a diverse array of MOF aerogels was successfully fabricated. These included ZIF‐8, MOF‐5, ZIF‐7, ZIF‐90, IRMOF‐3, MOF‐74(Zn), HKUST‐1, MOF‐74(Ni), NH_2_‐MIL‐53, MIL‐101(Fe), and ZIF‐67, showcased in Figure 4c. The pseudomorphic transformation pathway involves slow gel dissolution, rapid nucleation, and moderate crystal growth, generally preserving the original structure of the gel network and resulting in highly porous MOF aerogels.

*Drying Process*: The drying process critically influences the properties and porosity of the final MOF aerogel products. This process involves removing the solvent from wet gels, allowing the solid framework to retain its porous structure without significant collapse. Drying methods for MOF aerogels can be categorized into two main types: supercritical drying and ambient pressure drying.^[^ ^88^ ^]^

The obtained wet gels derived from sol‐gel synthesis usually require supercritical CO_2_ drying to produce MOF aerogels without any collapse or shrinkage. Supercritical CO_2_ drying is performed by exchanging the applied solvent (mainly alcohols) with liquid CO_2_ and removing the CO_2_ at a condition above its critical point (31 °C, 7.38 MPa). Using this drying approach, the aerogels finally feature high surface area, hierarchical porosity and low density. In contrast, ambient pressure drying primarily yield MOF xerogels, such as ZIF‐8,^[^ ^99^ ^]^ ZIF‐67,^[^ ^100^ ^]^ UiO‐66 (Figure 3d),^[^ ^91^ ^]^ MIL‐100(Fe),^[^ ^101^ ^]^ and MIL‐100(Al)^[^ ^101^ ^]^). This is due to capillary tension at the solid–liquid–vapor interface, which can lead to structural shrinkage or collapse.

In summary, the synthesis of MOF aerogels involves several critical factors. Precursor selection is paramount, with various metal salts (e.g., Fe, Cr, Cu, Al) and organic linkers (e.g., H_3_BTC, H_2_BDC) serving as building blocks. The choice of precursors significantly influences the final structure, porosity, and properties of the MOF aerogels. The solvent system is another crucial consideration. Common solvents include DMF, EtOH, and water, with some MOF aerogels requiring a mixture of solvents for optimal synthesis. Gelation conditions play a vital role in the process, with pH levels or gelation agent influencing both the gelation process and final structure. Temperature ranges from ambient to hydrothermal conditions (>100 °C), while gelation times vary from hours to days, depending on the specific MOF system. The drying method is essential for preserving the porous structure, with supercritical CO_2_ drying frequently used. Ambient pressure drying leads to xerogels, though some shrinkage may occur. MOF aerogels find their primary applications in gas uptake and water treatment, leveraging their high surface area and tunable pore structures. These materials show great promise in addressing environmental challenges and advancing sustainable technologies.

#### 2.1.2 The Formation of COF Aerogels

**Figure 5:**
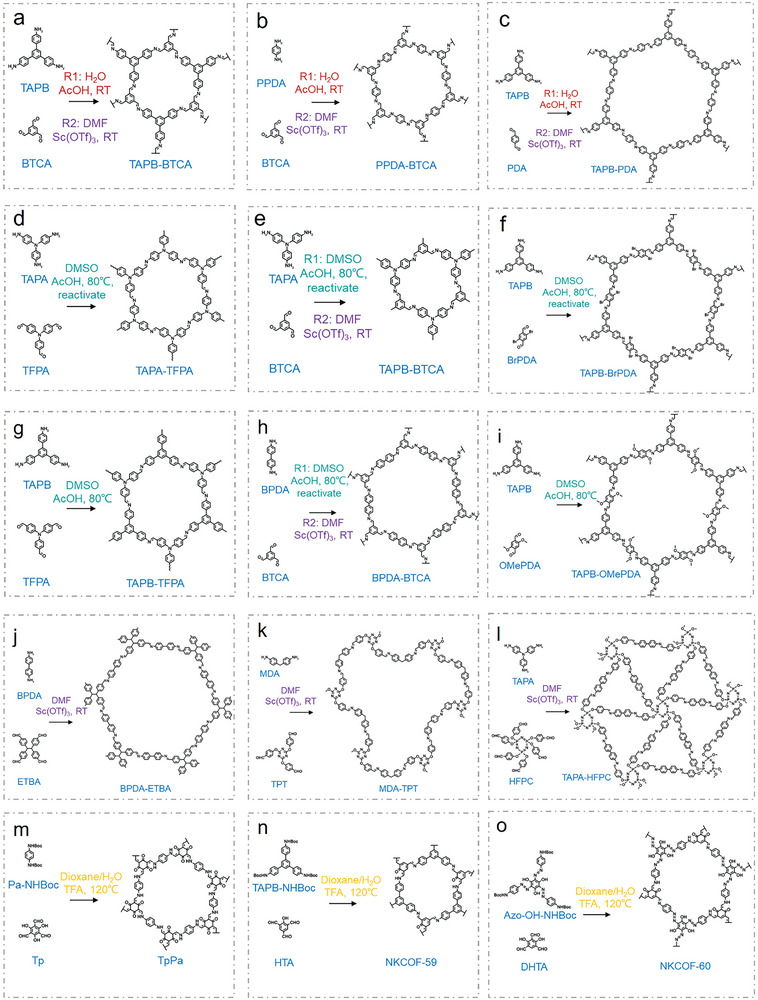
Synthetic routes of COF aerogels. a) TAPB‐BTCA,^[^ ^82^, ^84^ ^]^ b) PPDA‐BTCA,^[^ ^82^, ^84^ ^]^ c) TAPB‐PDA,^[^ ^82^, ^84^ ^]^ d) TAPA‐TFPA,^[^ ^83^ ^]^ e) TAPB‐BTCA,^[^ ^83^, ^84^ ^]^ f) TAPB‐BrPDA,^[^ ^83^ ^]^ g) TAPB‐TFPA,^[^ ^83^ ^]^ h) BPDA‐BTCA,^[^ ^83^, ^84^ ^]^ i) TAPB‐OMePDA,^[^ ^83^ ^]^ j) BPDA‐ETBA,^[^ ^84^ ^]^ k) MDA‐TPT,^[^ ^84^ ^]^ l) TAPA‐HFPC,^[^ ^84^ ^]^ m) TpPa,^[^ ^85^ ^]^ n) NKCOF‐59,^[^ ^85^ ^]^ and o) NKCOF‐60.^[^ ^85^ ^]^

COF aerogels, a new member in aerogel family, emerged after the first COF‐based hybrid aerogels were fabricated via encapsulation of COF particles into chitosan and graphene oxide (GO) aerogels.^[^ ^105^, ^106^ ^]^ Compared with MOF aerogels, COF aerogels are constructed entirely from light elements, such as C, N, O, H, and thus possess even lower density (**Figure** **5**). The preparation of COF aerogels involves a critical gelation process analogous to that used in the synthesis of MOF aerogels. Typically, the formation of COF gels is achieved through the cross‐linking of monomers within a catalyst‐enriched environment, under carefully controlled temperatures. As listed in **Table** **2** the synthesis parameters play a pivotal role in this process, with specific attention to the monomers, catalysts, solvents, and temperature conditions, all of which are crucial for successful gelation and the subsequent creation of COF aerogels.

**Table 2:** Synthesis conditions, physical properties, and suggested applications of COF aerogels prepared by the sol‐gel method.

COF aerogels	Monomer 1	Monomer 2	Solvent	Catalysis	*T*	Drying method	ρ_b_ [g cm^−3^]	*S*_BET_ [m^2^ g^−1^]	Applications	Refs.
TAPB‐BTCA	BTCA	TAPB	H_2_O	AcOH	RT	SD	0.0195	1146	Toluene uptake	2021^[^ ^82^ ^]^
PPDA‐BTCA	BTCA	PPDA					0.0208	677		
TAPB‐PDA	PDA	TAPB					0.0173	2535		
TAPA‐TFPA	TFPA	TAPA	DMSO	AcOH	80 °C	SD	0.031	186	Organic solvent absorption, dye adsorption, iodine uptake	2022^[^ ^83^ ^]^
TAPB‐PDA	PDA	TAPB					0.026	2273		
TAPB‐OMePDA	OMePDA	TAPB					0.032	2258		
TAPB‐BrPDA	BrPDA	TAPB					0.041	682		
TAPB‐TFPA	TFPA	TAPB					0.036	1626		
BPDA‐BTCA	BTCA	BPDA					0.031	1142		
TFPT‐HZ	TFPT	hydrazine hydrates	*o*‐dichlorobenzene	Sc(OTf)_3_	RT	FD	0.029	881	Thermal insulation	2022^[^ ^109^ ^]^
TPT‐HZ	TPT		EtOH				0.040	174		
COFA‐1	BDCA	TAPB	DMF	Sc(OTf)_3_	RT	FD	0.0224	254.6	Iodine uptake	2022^[^ ^84^ ^]^
COFA‐2	TFB	TAPB					0.0257	817.2		
COFA‐3	TFB	PPDA					0.0181	650		
NNS‐VCOF	TFPT	DTA	DMF	Sc(OTf)_3_	150 °C	FD	0.020	167.8	Thermal insulation, photocatalytic hydrogen evolution	2023^[^ ^110^ ^]^
TpPa	Pa‐NHBoc	Tp	Dioxane/H2O	TFA	120 °C	FD	0.0094	990	Solar desalination	2023^[^ ^85^ ^]^
DHTA‐Pa	Pa‐NHBoc	DHTA					0.0073	937		
TpBD	BD‐NHBoc	Tp					0.012	1283		
TpAzo	AzoNHBoc	Tp					0.0136	1625		
TAPB‐Tp	TAPB‐NHBoc	Tp	*o*‐dichlorobenzene/H_2_O				0.0152	1036		
NKCOF‐1	Azo‐OH‐NHBoc	Tp	Dioxane/H_2_O				0.018	418		
NKCOF‐58	TAPB‐NHBoc	DHTA					0.0143	720		
NKCOF‐59	TAPB‐NHBoc	HTA					0.0141	840		
NKCOF‐60	Azo‐OH‐NHBoc	DHTA					0.0162	322		
NKCOF‐61	Azo‐OH‐NHBoc	HTA	Dioxane/H_2_O				0.0159	637		
DT‐COF	TMTA	DFB	*o*‐dichlorobenzene/n‐butanol	KOH	90 °C	FD	0.0184	873.8	Adsorption, battery separator, piezoresistive sensing	2024^[^ ^111^ ^]^

*Monomers*: ICmine‐bond based COFs linked by aldehyde‐ and amine‐monomers have been first explored for their potential in aerogel formation. As a milestone in 2021 (Figure 3f), Zamora and colleagues successfully synthesized a series of crystalline imine‐based COF aerogels through a sol‐gel transition, followed by solvent exchange and supercritical CO_2_ drying processes.^[^ ^82^, ^107^ ^]^ In brief, COF wet gels were formed by cross‐linking of aldehyde‐ and amine‐monomers in the presence of glacial acetic acid (AcOH) aqueous solution at room temperature. Additional incubation for 5 d is needed for enhancing their porosity and crystallinity. After implementing a solvent exchange with a gradient from tetrahydrofuran to ethanol, the process was followed by supercritical CO_2_ drying. Porous imine‐based COF aerogels, characterized by low densities ranging from 0.017 to 0.021 g cm^−3^, were successfully obtained. Concerning the gelation mechanism, the authors explained that AcOH enable the establishment of new imine condensation bonds at the grain boundaries between the COF nanolayers. This process leads to the aggregation of these particles and the consequent formation of a 3D gel structure. The monolithic TAPB‐PDA‐COF, PPDA‐BTCA‐COF, and TAPB‐PDA‐COF aerogels (Figure 5a–c), synthesized from corresponding aldehyde monomers (1,3,5‐benzenetricarbaldehyde, BTCA, or terephthalaldehyde, PDA) and amine monomers [1,3,5‐tris(4‐aminophenyl)benzene, TAPB, or 1,4‐diaminobenzene, PPDA], exhibit a hierarchically organized porous structure. This structure encompasses micro‐, meso‐, and macro‐pores, contributing to an overall porosity of ≈99%. This value is marginally higher than that of traditional silica aerogels due to the intrinsic porosity of the COFs, which enhances the overall porosity by ≈3%–5%. Additionally, the BET surface areas of these COF aerogels are comparable to those produced through solvothermal methods, affirming the quality of the aerogels formed. Moreover, all aerogels behaved elastically under strain below 25%–35%. Furthermore, this strategy has proven effective for synthesizing COF aerogel/nanomaterial composites with multiple functions, such as Fe_3_O_4_@reduced graphene oxide (rGO)/COF aerogel.^[^ ^82^ ^]^ This advancement in COF aerogels opens the door to a wide array of novel COF aerogels and associated composites, expanding their potential applications and functionalities.

Beta‐ketoenamine‐linked COFs, a subclass of imine‐based COFs notable for their enhanced stability, have been investigated for aerogel synthesis. These COFs undergo a biphasic transformation process. Initially, a reversible reaction facilitates the formation of a well‐ordered crystalline framework via the classical Schiff‐base reaction. This is followed by an irreversible change from the enol–imine (OH) form to the more stable keto–enamine (NH) configuration. Significantly, this permanent shift to the keto‐enamine structure bolsters the COF's resilience, preserving its crystalline integrity even under rigorous conditions such as boiling water or in the presence of potent acids or bases.^[^ ^108^ ^]^ Zhang's research group has recently introduced a versatile synthetic approach for beta‐ketoenamine‐linked COF gels, which involves the modification of amine monomers with the *tert*‐butoxycarbonyl (Boc) protective group. The incorporation of the Boc group slows the reaction kinetics and promotes the formation of hydrogen bonds among COF clusters, aiding in gelation. Through this method, ten distinct types of COF gels, including TpPa, DHTA‐Pa, TpBD, TpAzo, TAPB‐Tp, and NKCOF series (−1, −58, −59, −60, and −61), were crafted, each exhibiting high crystallinity and a porous architecture. These gels could be subsequently converted into COF aerogels via solvent exchange and lyophilization, solidifying their form while preserving the porous characteristics.^[^ ^85^ ^]^

*Catalysts*: Catalysts play an essential role in dictating the gelation rate of COF gels. Traditional usage of AcOH as a catalyst typically requires an extended period of undisturbed reaction.^[^ ^82^ ^]^ Given this fact, scandium trifluoromethanesulfonate (Sc(OTf)_3_) has been suggested as a more efficient catalyst for generating crystalline COF gels from amino monomers (TAPB, PPDA) and aldehyde monomers (BDCA, 1,4‐benzenedicarboxaldehyde; TFB, 1,3,5‐triformylbenzene) (Figure 5a–c,e,h,j–l). Remarkably, gelation using Sc(OTf)_3_ as the catalyst can occur within just 5 min at room temperature. This rapid formation contrasts sharply with the use of acetic acid, which under identical conditions, is unable to facilitate gel formation.^[^ ^84^ ^]^

In addition to AcOH and Sc(OTf)_3_ as gelation agents, trifluoroacetic acid (TFA) was also introduced as a catalyst. The amount of TFA used plays a crucial role in the formation and crystallinity of TpPa COF gels synthesized from 2,4,6‐trihydroxybenzene‐1,3,5‐tricarbaldehyde (Tp) and benzene‐1,4‐diamine (Pa‐NH_2_) monomers. The quality of the gels reaches an optimal level at a relatively low TFA content, where the reaction of the monomers is sufficient. However, higher amounts of TFA result in decreased crystallinity. It's worth noting that other acids, such as trifluoromethanesulfonic acid, *p*‐toluenesulfonic acid, and sulfuric acid, can also effectively promote the formation of COF gels. This fine balance in acid concentration highlights the importance of precise control over reaction conditions in the synthesis of high‐quality COF gels.^[^ ^85^ ^]^

Solvents: In addition to using water as the reaction solvent, DMSO was also selected to dissolve amino monomers such as TAPB, TAPA [tris(4‐aminophenyl)amine), BPDA ((1,1′‐biphenyl])‐4,4′‐diamine] and aldehyde monomers including PDA (terephthaldehyde), BrPDA (2,5‐dibromoterephthalaldehyde), OMePDA (2,5‐dimethoxyterephthalaldehyde), TFPA (tris(4‐formylphenyl)amine), and BTCA (1,3,5‐benzenetricarboxaldehyde) (Figure 5d–i). Gelation took place within just 1 min of adding the acid catalyst. After complete reaction, the hazy gels were solvent exchanged and dried under supercritical CO_2_ to obtain final crystalline COF aerogels. It is worth noting that this gelation and supercritical CO_2_ drying route could so far only produce two crystalline COF aerogels (TAPB‐OMePDA and TAPB‐TFPA), thus additional reactivation and supercritical CO_2_ drying processes are necessary for tuning TAPA‐TFPA, TAPB‐PDA, TAPB‐BrPDA, and BPDA‐BTCA amorphous aerogels into their crystalline COFs aerogel counterparts (Figure 5d–f,h).^[^ ^83^ ^]^ Similarly, the BET surface areas of the crystalline COF aerogels are comparable or even larger than their respective powders and the densities are in the range of 20–40 mg cm^−3^.

Furthermore, it has been proven that the presence of water and organic solvents, which contain hydrogen‐bond donors and acceptors, is critical for producing high‐quality COF gels with Boc‐modified amine monomers. For instance, in the absence of water, the resulting product was a powdery solid instead of a gel. Additionally, successful synthesis of COF gels was accomplished using solvents such as chlorobenzene, *o*‐dichlorobenzene, tetrachloroethane, tetrahydrofuran, and *n*‐butanol. However, attempts to create gels with mesitylene and *n*‐heptane did not yield successful results.^[^ ^85^ ^]^

In summary, the precursors for COF aerogels vary depending on the framework type. Imine‐based COFs, which are the most extensively studied, typically utilize aldehydes and amines, offering exceptional versatility in precursor selection and functionalization. Hydrazone‐based COFs, on the other hand, employ hydrazides and aldehydes. Common organic solvents used in COF aerogel synthesis include DMF, DMSO, and EtOH. Some syntheses incorporate mixed systems, combining water with organic solvents. Catalysts play a crucial role in the formation of COF aerogels. For imine and hydrazone‐based COFs, catalysts such as AcOH, Sc(OTf)_3_, and TFA are frequently used. Gelation temperatures may range from room temperature to ≈150 °C, while gelation times can span from several hours to multiple days, depending on the specific synthesis requirements. Supercritical CO_2_ drying produces aerogels with high surface area and porosity, while freeze‐drying results in cryogels with hierarchical pore structures. COF aerogels have found widespread applications in various fields, including gas uptake, water treatment, catalysis, thermal insulation, and solar desalination, demonstrating their versatility and potential for addressing diverse technological challenges.

#### 2.1.3 Comparison of MOF and COF Aerogels

MOF and COF aerogels are both classes of porous materials characterized by high surface areas and low densities. While they share some similarities, there are significant differences in their composition, synthesis, and properties when prepared by the sol‐gel method.

MOF aerogels consist of metal ions or clusters coordinated to organic ligands, incorporating both inorganic and organic components. Their structure is formed by metal–ligand coordination bonds. The synthesis process involves dissolving metal salts and organic ligands in a solvent, allowing coordination bonds to form between metal ions and ligands. Gelation occurs through the formation of a 3D network, followed by solvent exchange and supercritical drying to obtain the final aerogel structure. Due to their weaker coordination bonds, MOF aerogels are generally less stable and more susceptible to degradation under harsh conditions. The density can be fine‐tuned by selecting different metal ions and ligands. Their pore sizes can range from microporous to mesoporous, and they can incorporate various functionalities through both metal centers and organic ligands. These unique properties make MOF aerogels promising candidates for applications in catalysis, gas storage, and separation processes.

In contrast, COF aerogels are composed entirely of light elements, typically C, N, O, H, and consist solely of organic components. The framework is formed through covalent bonds between organic building blocks. The synthesis process begins with dissolving organic monomers in a suitable solvent, followed by condensation reactions that form covalent bonds between monomers. As the covalent network expands, gelation takes place, and the aerogel is then obtained through solvent exchange and supercritical drying. COF aerogels are characterized by their higher chemical and thermal stability due to strong covalent bonds, which make them more resistant to harsh conditions. While their tunability is limited to organic building blocks, diverse structures can be achieved through the use of different organic monomers. The absence of metal ions in COF aerogels results in lower densities compared to MOF aerogels, with density primarily dependent on the organic building blocks used. Their functionality is mainly derived from these organic components, rendering COF aerogels particularly suitable for applications in gas storage, separation processes, and optoelectronics.

### 2.2 Templating Methods

Templates such as ice,^[^ ^112^, ^113^ ^]^ emulsions,^[^ ^114^, ^115^, ^116^, ^117^ ^]^ gas bubbles,^[^ ^118^, ^119^ ^]^ or NaCl crystals^[^ ^120^ ^]^ have been utilized for fabricating MOF/COF aerogels or foams that contain macropores alongside intrinsic micropores. This approach allows precise control over the size, shape, and structure of the resulting MOF/COF gels, facilitating the development of specific morphologies that can enhance performance in various applications. For example, a porous HKUST‐1 monolith has been prepared by ice‐templating, utilizing the directional freezing of a warm DMSO‐precursor solution followed by freeze‐drying. The macropores display a bimodal size distribution, featuring peaks at around 10 µm for the aligned structures and 0.4 µm for these within the walls. This alignment in the porous structure potentially offers a low pressure drop, which is beneficial for applications in column separation.^[^ ^112^ ^]^ The structural robustness of MOF networks can be enhanced, making them more resilient to mechanical stress. Chen et al. prepared NiMn‐MOF nanobelts using a solvothermal method first, which were then dispersed in water, followed by slow freezing with liquid nitrogen and freeze‐drying to obtain MOF aerogels with a density of 3.6 mg cm^−3^. This MOF aerogel could restore its original shape after a 50% strain and keep structure stability after 2000 compression–resilience cycles.^[^ ^113^ ^]^ Such a versatile ice‐template approach is a promising avenue of research toward further design and development of functional materials based on low‐dimensional MOF and COF building blocks.^[^ ^121^ ^]^

Moreover, a MOF‐stabilized high‐internal‐phase emulsion (HIPE) was used to derive ultralight MOF aerogels based on the building blocks of 0D Cu_3_(BTC)_2_ nanoparticles, 1D Mn_3_(BTC)_2_ nanowires, and 2D Ni(BDC) nanosheets.^[^ ^114^ ^]^ Typically, water, diethyl ether and MOF powders were first mixed for emulsification. After the emulsion fully separated into two phases, the gel‐like HIPEs, characterized by high viscosity and low MOF concentration (less than 3.0 wt%) were stepwise dried for producing an intact and highly porous aerogel. The resulting Cu_3_(BTC)_2_ aerogel shows a high macropore volume of 9.0 cm^3^ g^−1^, promoting the diffusion of molecules into the pores and improving the adsorption performance of dyes.

Pioneering work by Banerjee et al. applied a gas‐foaming protocol to prepare a series of COF foams.^[^ ^118^ ^]^ Various amines building units reacted with *p*‐toluene sulfonic acid (PTSA) and triformylphloroglucinol (Tp) to form 2D crystalline network. The reaction between sodium bicarbonate (NaHCO_3_) and PTSA led to the continuous release of CO2, facilitating the formation of heterogeneous pores. After lyophilization and heating, lightweight and hierarchically porous COF foams have been achieved. TpBD‐Me2 foam contains a broad pore size distribution ranging from 10 to 150 nm and 2.3 to 390 µm as well as a high surface area of 797 m^2^ g^−1^. The interconnected pore structures facilitate rapid adsorption and subsequent diffusion of pollutants through the pore channels, enhancing their removal efficiency. Furthermore, NaCl crystals were also applied as pore‐forming template for preparing TpBD‐COF foams, which can be released easily by water after polymerization of TP and BD under the catalysis of PTSA. The pore structure of TpBD‐COF foam is highly dependent on the dosage of NaCl. The optimized TpBD foam features abundant macropore and mesopore as well as large specific surface area (≈700 m^2^ g^−1^), which was demonstrated to be a promising adsorbent for the efficient removal of sulfamerazine in aqueous solution.^[^ ^120^ ^]^ Additionally, the method can be used to create a variety of structures, including monolithic, granular, and fiber forms, enabling customization for specific applications in fields such as drug delivery, catalysis, and energy storage. Furthermore, the templating method can be easily scaled up for industrial production, making it feasible for manufacturing larger quantities of MOF/COF materials compared to other methods. Overall, the templating method is a versatile and effective approach for synthesizing high‐performance MOF/COF aerogels, tailoring their properties for specific applications across various fields.

## 3 MOF/COF Hybrid Aerogels

Hybrid aerogels based on MOF/COF materials, when integrated with (bio)polymers, graphene, and MXenes, offer a range of advantages due to the combined properties of the frameworks and the additives. First, incorporating (bio)polymers or graphene can greatly improve the mechanical strength and flexibility of MOF/COF‐based aerogels, enhancing their durability and making them more suitable for structural applications.^[^ ^122^ ^]^ Second, the integration with polymers or other materials also allows for the adjustment of pore size, surface area, and connectivity, optimizing these aerogels for functions like adsorption and catalysis.^[^ ^123^ ^]^ Third, the addition of graphene and MXenes, noted for their excellent electrical conductivity, can be used to enhance applications in energy storage, sensors, and flexible electronics.^[^ ^105^ ^]^ Finally, incorporating biopolymers supports sustainable practices, as they are often sourced from renewable resources, thus reducing the environmental impact of aerogel production. Therefore, in addition to pure MOF/COF aerogels, this review also covers two general approaches for the integration of MOFs/COFs with molecular precursors and nanoscale materials such as, 0D nanoparticles, 1D nanofibers/nanotubes, or 2D nanosheets to produce 3D MOF/COF‐based aerogels.

**Figure 6:**
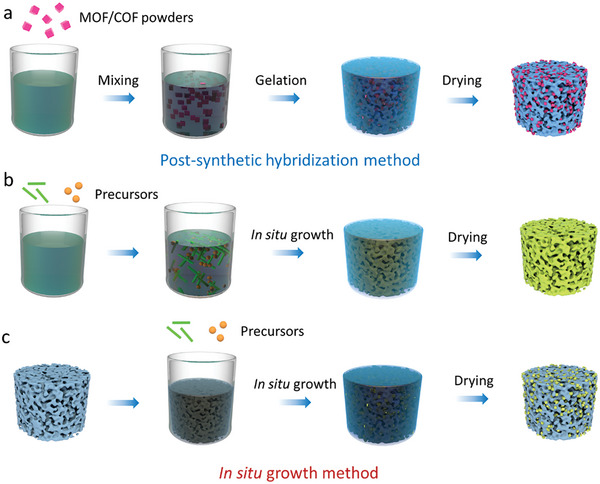
MOF/COF hybrid aerogels fabricated by postsynthetic hybridization and in situ growth methods.

One approach is the postsynthetic hybridization method (**Figure** **6**a), where a sol solution of molecular precursors and colloidal dispersions of nanoscale materials, along with preformed MOF/COF powders, are combined into colloidal suspensions via covalent or noncovalent interactions. The following gelation traps MOF/COF particles inside the gel matrix. After further supercritical drying or freeze‐drying, hierarchically MOF/COF‐containing hybrid architectures can be obtained. The other approach is the in situ growth method. Here, either the MOF/COF precursors are introduced into a sol solution of low‐dimensional building blocks to form gels together (Figure 6b), or preformed 3D frameworks are immersed into a solution of MOF/COF precursors (Figure 6c), which then assemble within the framework. Thus, in the latter two cases, MOFs/COFs are grown in situ as crystals or films on the supporting materials during the reaction and the following drying step yield 3D hybrid structures.

**Figure 7:**
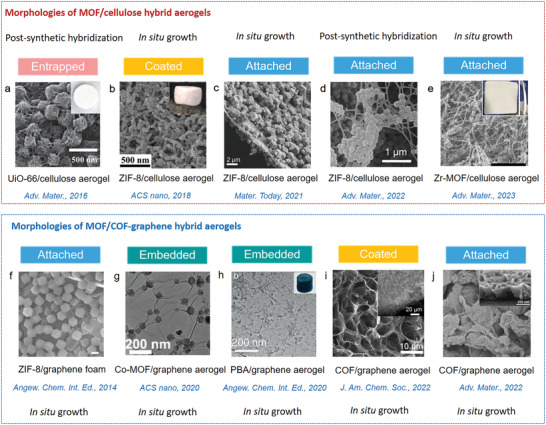
a–e) Morphologies of MOF/cellulose hybrid aerogels. a) Entrapped. UiO‐66/ cellulose aerogel. Reproduced with permission.^[^ ^124^ ^]^ Copyright 2016, Wiley‐VCH. b) Coated. ZIF‐8/ cellulose aerogel. Reproduced with permission.^[^ ^125^ ^]^ Copyright 2018, American Chemical Society. c–e) Attached. ZIF‐8/cellulose aerogel. Reproduced under the CC BY‐NC‐ND license.^[^ ^126^ ^]^ Copyright 2021, Elsevier. ZIF‐8/cellulose aerogel. Reproduced with permission.^[^ ^122^ ^]^ Copyright 2022, Wiley‐VCH. Zr‐MOF/cellulose aerogel. Reproduced with permission.^[^ ^123^ ^]^ Copyright 2023, Wiley‐VCH. f–h) Morphologies of MOF/COF‐graphene hybrid aerogels. f) Attached. ZIF‐8/graphene foam. Reproduced with permission.^[^ ^127^ ^]^ Copyright 2014, Wiley‐VCH. g) Embedded. Co‐MOF/graphene aerogel. Reproduced with permission.^[^ ^128^ ^]^ Copyright 2020, American Chemical Society. h) PBA/graphene aerogel. Reproduced with permission.^[^ ^129^ ^]^ Copyright 2020, Wiley‐VCH. i,j) Morphologies of COF/graphene hybrid aerogels. Reproduced with permission.^[^ ^130^ ^]^ Copyright 2022, American Chemical Society. Reproduced with permission.^[^ ^131^ ^]^ Copyright 2022, Wiley‐VCH.

Depending on the reaction conditions used, different morphologies are obtained as shown in **Figure** **7** with examples from the literature including attached, embedded, bulged (penetrated), agglomerated or coated MOF/COF composite structures. The morphologies of 3D MOF/COF hybrid aerogels are related to the interfacial compatibility of the two components and are important to assess the performance of the aerogels for specific applications. For example, MOF crystals can be incorporated between cellulose fibers via a postsynthetic hybridization method (Figure 7a). Typically, attached and coated morphologies of MOF/cellulose and MOF/graphene aerogels are achieved through in situ growth, making them ideal for applications such as adsorption, filtration, catalysis, and sensing (Figure 7b–f). The MOF crystals are embedded within the aerogel thanks to the excellent dispersion of the MOF precursor in the processing solution, which benefits energy applications (Figure 7g,h). In the case of COF/graphene aerogels, COF crystals are coated or attached to a conductive substrate, enhancing electron transport capabilities for energy and catalysis (Figure 7i,j). Therefore, controlling these morphologies is crucial for developing high‐performance 3D MOF/COF hybrid aerogels.

### 3.1 MOF‐Oxide Aerogels

**Figure 8:**
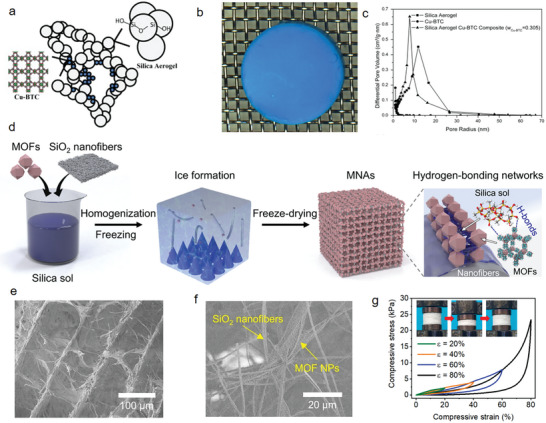
a) Scheme and b) photograph of Cu‐BTC‐silica aerogel composites. c) Pore size distribution of Cu‐BTC, silica aerogel and Cu‐BTC‐silica aerogel. Reproduced with permission.^[^ ^132^ ^]^ Copyright 2013, Elsevier. d) Fabrication process, e,f) SEM images, and g) mechanical properties of MOF/SiO_2_ fiber aerogel. Reproduced with permission.^[^ ^142^ ^]^ Copyright 2023, Springer Nature.

Integrating MOF nanocrystals within 3D porous oxide aerogels is an effective strategy to achieve pore size from micropores to mesopores thus to enhance the mass transport as well as promoting gas separation and catalysis. MOF nanocrystals can act as second phase to be incorporated into SiO_2_^[^ ^132^, ^133^, ^134^, ^135^ ^]^ or TiO_2_^[^ ^136^, ^137^, ^138^, ^139^, ^140^, ^141^ ^]^ aerogels. This can be realized through sol‐gel process of the oxide in presence of MOF nanocrystals followed by supercritical drying. It is critical to control the mixing time of presynthesized MOF nanocrystals during the sol‐gel process for the formation of bulk aerogels and preservation of the MOF structure. As an example, Cu‐BTC nanocrystals were initially mixed with a silica precursor solution. After hydrolysis and condensation reaction of the silica precursor, solvent exchange and supercritical drying, a blue monolith composed of uniformly dispersed microporous domains of Cu‐BTC embedded within the mesoporous SiO_2_ aerogel network is obtained (**Figure** **8**a,b).^[^ ^132^ ^]^ The composite aerogel has a broader pore size distribution with an average pore radius between that of Cu‐BTC and SiO_2_ aerogel (Figure 8c), achieving a surface area of 1138 m^2^ g^−1^. A further development in Cu‐BTC/SiO_2_ aerogel relates to the introduction of Cu‐BTC nanocrystals into a presynthesized SiO_2_ sol. This synthetic approach results in a higher surface area of 1655 m^2^ g^−1^.^[^ ^134^ ^]^ Inspired by the work of MOF/SiO_2_ aerogels, MOF/TiO_2_ aerogels have been synthesized in a similar approach with prospective applications for dye‐sensitized solar cell^[^ ^141^ ^]^ and supercapacitors.^[^ ^139^ ^]^ To overcome the intrinsic brittleness of 0D granular‐structured oxide aerogels, 1D silica nanofiber assembled aerogels have been developed with incorporating MOF particles. An aligned lamellar structure was achieved through an additional ice‐templating approach resulting in the strong adhesion dominated by the hydrogen bond between MOFs and SiO_2_ nanofibers (Figure 8d–f), endowing the aerogel with high porosity, low filtration resistance and elasticity (Figure 8g). This type of MOFs‐based aerogels shows potential in the field of outdoor emergency life‐saving devices against chemical warfare agents threats.^[^ ^142^ ^]^

### 3.2 MOF/COF‐(Bio)polymer Hybrid Aerogels

#### 3.2.1 Postsynthetic Hybridization Strategy

**Figure 9:**
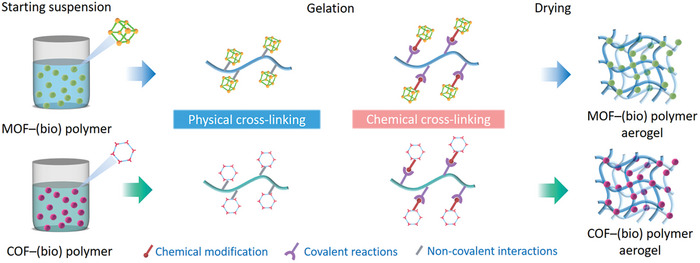
Typical preparation procedure of MOF/COF‐(bio)polymer aerogels by the postsynthetic hybridization method.

MOF/COF‐(bio)polymer aerogels can be fabricated by a postsynthetic hybridization strategy (**Figure** **9**), starting from a suspension of presynthesized MOF/COF nanocrystals within solutions of (macro)molecules. After the gelation, which can be initiated by chemical routes, e.g., by condensation and cross‐linking of monomeric precursors,^[^ ^143^, ^144^, ^145^, ^146^ ^]^ physical routes in which gelation is promoted by noncovalent interactions (hydrogen bonding and electrostatic interactions),^[^ ^124^, ^147^, ^148^, ^149^, ^150^, ^151^ ^]^ or even by solidification of the suspension by freeze casting,^[^ ^122^, ^152^, ^153^, ^154^ ^]^ MOF/COF‐(bio)polymer hybrid aerogels with porous skeletons are finally achieved by supercritical or freeze drying. In the initial mixing process, the surface chemical modification of the MOF/COF nanocrystals^[^ ^144^, ^146^ ^]^ and the (macro)molecules,^[^ ^106^, ^155^ ^]^ as well as the amount of MOF/COF^124^ play a critical role for the subsequent gelation behavior and the final morphology of MOF/COF‐(bio)polymer aerogels.

MOFs and COFs both exhibit the capacity for intense chemical and physical interactions with (bio)polymers, though the nature and strength of these interactions vary significantly. MOFs offer greater versatility in potential interactions and can markedly alter (bio)polymer properties, albeit potentially at the cost of composite stability. Conversely, COFs can establish stronger and more stable covalent bonds with (bio)polymers, potentially resulting in more robust composite materials with more predictable properties. The structural differences between MOFs and COFs also influence their behavior in (bio)polymer matrices. MOFs, with their 3D structure and potential for stronger interactions, may achieve superior dispersion in certain (bio)polymer matrices. In contrast, COFs, typically existing as 2D or layered structures, may encounter challenges in achieving uniform dispersion within some (bio)polymer systems. These distinctions in bonding mechanisms, stability, and structural characteristics have profound implications for the design, fabrication, and application of (bio)polymer‐based aerogels incorporating MOFs or COFs.

**Figure 10:**
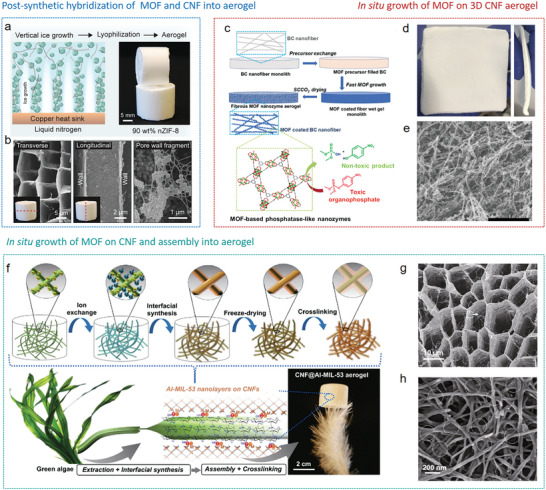
The fabrication processes and microstructures of MOF/cellulose aerogels prepared by different strategies. a) Postsynthetic hybridization of ZIF‐8 and CNF into aerogel by freeze‐casting, and b) microstructures of ZIF‐8/CNF aerogel. Reproduced with permission.^[^ ^122^ ^]^ Copyright 2022, Wiley‐VCH. c) In situ growth of MOFs on a 3D CNF aerogel. d) Photos and e) microstructure of Zr‐MOF/BC aerogel. Reproduced with permission.^[^ ^123^ ^]^ Copyright 2023, Wiley‐VCH. f) In situ growth of Al‐MIL‐53 on cellulose nanofibers and assembly into aerogels. g,h) Microstructures of aligned porous Al‐MIL‐53‐coated CNF aerogels. Reproduced with permission.^[^ ^180^ ^]^ Copyright 2020, Springer Nature.

Simply mixing MOF/COF crystals with unmodified (macro)molecules usually leads to low loadings and inhomogeneous distribution of MOF/COF crystals within the aerogel matrix, yielding weak mechanical properties. An improved compatibility can be achieved with functional groups on the (bio)polymer building blocks which provide attractive interaction with the MOF/COF crystals. For instance, ZIF‐8, UiO‐66, or MIL‐100(Fe) particles were mixed with celluloses that have been functionally modified at right angles, including aldehyde‐functionalized cellulose nanocrystals (CHO–CNCs) and hydrazide‐functionalized carboxymethyl cellulose (NHNH_2_–CMC). The latter was applied to form hydrazone crosslinks and a stable colloidal suspension. Consequently, well dispersed MOF particles with high loadings (50 wt%) within the cellulose aerogel are observed, which have been applied as absorbents for water purification.^[^ ^124^ ^]^ Rostami et al. presented a strategy to fabricate aerogels with up to 90 wt% nano ZIF‐8 loading by unidirectional freeze‐casting, using cellulose nanofibrils as the polymeric host (**Figure** **10**a). The resulting ZIF‐8/CNF hybrid aerogel displays parallel tubular pores with the pore walls adorned with ZIF‐8 particles (Figure 10b). Attributed to the strong interfacial adhesion of the interconnected networks, the aerogels are chemically stable and thus have been tested for diverse applications, e.g., water purification, gas adsorption and separation, and fireproof insulations.^[^ ^122^ ^]^

In addition to conventional porous monoliths, highly porous MOF beads have been created using a double‐cross‐linked network composed of poly(acrylic acid) (PAA), sodium alginate, and calcium ions (Ca^2+^). The incorporation of PAA and Ca^2+^ facilitates cross‐linking with alginate chains through hydrogen bonding and ionic interactions, thereby enhancing the stability of the beads and improving their effectiveness in liquid separations. Such cross‐linked networks were applied as host for a wide range of MOFs.^[^ ^156^ ^]^ Another example is COF–chitosan aerogel, for which 1,4‐butanediol diglycidyl ether was used as the cross‐linker during the gelation process to form stable networks based on ─NH─CH_2_─CH(OH)─ linkages, which formed through amino‐epoxy reaction. Robust COF–chitosan aerogels with high COF loadings (50 wt%) could be prepared, which are lightweight (5.6–6.9 mg cm^−3^) and machinable.^[^ ^106^ ^]^

All these examples show aerogels in which non‐covalent interactions are present between MOF/COF and the polymer phase. To improve the binding force between MOF/COF particles and (bio)polymer aerogels and avoid their agglomeration, MOF/COF‐(bio)polymer aerogels have been fabricated by covalent coupling of MOF/COF particles with the gel polymers.^[^ ^144^, ^146^ ^]^ For instance, UiO‐66‐ polyimide (PI) aerogels were synthesized by covalent interactions. In detail, anhydride‐functionalized UiO‐66‐NH_2_ particles were chemically linked to PI monomers via a single‐step amidation polymerization process. This was then succeeded by freeze‐drying and thermal imidization steps.^[^ ^146^ ^]^

Compared to MOFs, the abundant surface functional groups on COFs are conducive to the formation of COF aerogels based on covalently cross‐linking. For example, considering the aldehyde groups and hydrazine groups present on azine‐linked COF surfaces, a ring‐opening polymerization of amine groups with epoxy groups has been adopted to construct COF‐based aerogels.^[^ ^157^ ^]^ The resulting hierarchically porous COF monoliths exhibited pores ranging from 0.43 to 3.51 µm, showing promising performance in the removal of bisphenol A from water. Furthermore, a composite aerogel of COF and chitosan was created through the chemical crosslinking of an allyl‐imidazolium ionic liquid‐coated COF with a thiol‐functionalized chitosan binder, utilizing a photoinduced thiol–ene coupling reaction (Figure 9e). Notably, these COF‐chitosan aerogels retain processability even when loaded with up to 80 wt% of the COF.

#### 3.2.2 In Situ Growth Method

The most common approach for the development of MOF‐(bio) polymer aerogels is by in situ growth of MOFs on the preformed 3D porous (bio) polymer skeleton. To date, various MOFs (ZIF‐67,^[^ ^158^, ^159^, ^160^ ^]^ ZIF‐8,^[^ ^161^, ^162^ ^]^ UiO‐66,^[^ ^155^, ^163^, ^164^, ^165^ ^]^ MIL‐53,^[^ ^166^ ^]^ MIL‐88,^[^ ^167^ ^]^ MIL‐100(Fe),^[^ ^168^ ^]^ MIL‐101,^[^ ^167^ ^]^ Cu‐BDC,^[^ ^169^, ^170^ ^]^ HKUST‐1,^[^ ^171^ ^]^ PCN‐224,^[^ ^172^ ^]^ Ni‐HITP,^[^ ^173^ ^]^ and MOF‐525^[^ ^174^ ^]^) and COFs (COF‐DTF,^[^ ^175^ ^]^ COF‐TPDA‐2,3Dha,^[^ ^176^ ^]^ and COF‐SO_3_H^[^ ^177^ ^]^) have been in situ grown on untreated or treated bio(polymer) aerogels based on two‐step (metal ion‐first or organic linker‐first) or one‐pot (MOF/COF precursor solutions) immersion strategies, which are divided according to the addition sequence of metal ions and organic ligands.

In a recent study that used a metal ion‐first, two‐step strategy, bacteria cellulose (BC) aerogels were initially immersed in a solution containing metal ions. The extensive hydroxyl groups on BC nanofibers enabled their efficient absorption of metal ions via electrostatic interactions. This was followed by the nucleation of MOF nanoparticles within an organic ligand solution. The as‐prepared ZIF‐8 and UiO‐66 nanoparticles were interwoven with crystalline BC nanofibers, resulting in a distinctive string‐beaded morphology. Metal ions (Zn^2+^, Cu^2+^, and Co^2+^) can also interact with carboxyl functionalized CNFs, transforming the individual fibers into a homogeneous fibrous hydrogel (CNFs‐M^2+^).^[^ ^125^, ^178^ ^]^ Subsequently, these metal ions facilitate the growth of MOF crystals around the CNFs in the presence of suitable ligands. The resulting MOF aerogels feature hierarchically porous microstructures, and are characterized by their ultralight weight, flexibility, and mechanical robustness. This strategy has also been demonstrated in the fabrication of aramid nanofibril aerogel supported MOFs for efficient adsorption and interception.^[^ ^179^ ^]^

The organic ligand‐first strategy was developed to engineer MOF films on CMC aerogel walls. Initially, the organic ligand is strategically “pre‐seeded” onto the aerogel's internal walls. Subsequently, a minimal quantity of metal ions solution is delicately sprayed onto the pretreated aerogel surface. This solution then diffuses uniformly along the aerogel walls, creating a seamless, thin layer. This layer effectively confines and catalyzes the nucleation process, facilitating the uniform formation of MOF films. This versatile fabrication approach has been successfully applied to synthesize a variety of MOF/aerogel composites.^[^ ^169^ ^]^ Such hierarchical aerogels have shown to be catalytic active and stable in both liquid phase CO_2_ cycloaddition to produce cyclic carbonates and for electrochemical catalysis.

Instead of seeding metals or linkers consecutively, a one‐pot strategy was developed to integrate Zr‐MOF into a flexible and processable BC aerogel substrate. The cellulose nanofibers, abundant in hydroxyl groups, combined with a high concentration of MOF precursors, enhance rapid nucleation and enable swift growth of MOF coatings on the fibrous aerogel substrate. The formed hierarchical structure enables accessibility to possible catalytic active sites within the MOF (Figure 10c–e).^[^ ^123^ ^]^ This one‐pot approach was also employed for growing a PCN‐224(M) (M = Fe, Ni, Co, and Ru) on acid pretreated melamine foams. In this case MOF nanoparticles decorated the fibers like a ball‐and‐stick model.^[^ ^172^ ^]^

Besides in situ growth of MOF on preformed 3D porous aerogels, another attractive approach involves in situ growth of MOF on biopolymer nanofibers building blocks, which are then assembled into 3D porous structures by freeze casting.^[^ ^180^, ^181^ ^]^ This method yields ordered cellular aerogels with highly uniform MOF layer‐coated biopolymer struts. Reasonable interface design is the prerequisite for in situ growth of MOF on biopolymer building blocks nanofibers. In the production of aligned porous Al‐MIL‐53‐coated CNF aerogels, the carboxylated CNFs first engaged in ion exchange with Al^3+^ to form a CNF‐COO^−^‐Al^3+^ complex. Subsequently, the interfacial synthesis of Al‐MIL‐53 nanolayers on the CNFs was initiated by coordination between the Al^3+^ bound to the CNFs and a disodium terephthalate (Na_2_BDC) linker. Following freeze casting and drying, the aerogel was further immersed in an aqueous solution of precursors to promote the nucleation of Al‐MIL‐53 onto the nanofibers (Figure 10f). Consequently, the aerogel presented an interconnected cellular network structure, with the network walls constructed by entwining and interweaving of the Al‐MIL‐53 coated nanofibers (Figure 10g,h). The as‐prepared aerogels shown elasticity, thermal insulation, moisture resistance, and good fire retardancy.^[^ ^180^ ^]^

### 3.3 MOF/COF‐Graphene Hybrid Aerogels

The development of 3D MOF/COF‐graphene hybrid aerogels represents a significant advancement in the field of materials science, especially in the areas of energy storage, catalysis, sensing, and environmental remediation.^[^ ^182^, ^183^ ^]^ These aerogels combine the high surface area, tunability, and selective adsorption properties of MOFs and COFs with the outstanding mechanical strength, electrical conductivity, and thermal stability of graphene. The 3D porous structure of these aerogels further enhances their performance by providing a highly accessible surface area and facilitating the diffusion of molecules or ions. For example, the porous nature and high surface area of 3D MOF/COF‐graphene aerogels make them excellent catalysts and catalyst supports. These materials facilitate the dispersion of catalytic sites and enhance the accessibility of reactants, resulting in increased catalytic activity and selectivity for various reactions, including those relevant to energy conversion and environmental remediation.^[^ ^184^, ^185^ ^]^

The fabrication of 3D MOF/COF‐graphene hybrid aerogels usually involves self‐assembly processes where GO serves as a starting material. The GO sheets are typically reduced and assembled into a 3D structure through hydrothermal or solvothermal treatments, sometimes aided by freeze‐drying techniques to preserve the porous structure.^[^ ^186^, ^187^, ^188^ ^]^ MOFs or COFs precursors are incorporated during or after the assembly of the graphene framework, resulting in a well‐integrated hybrid structure.

**Figure 11:**
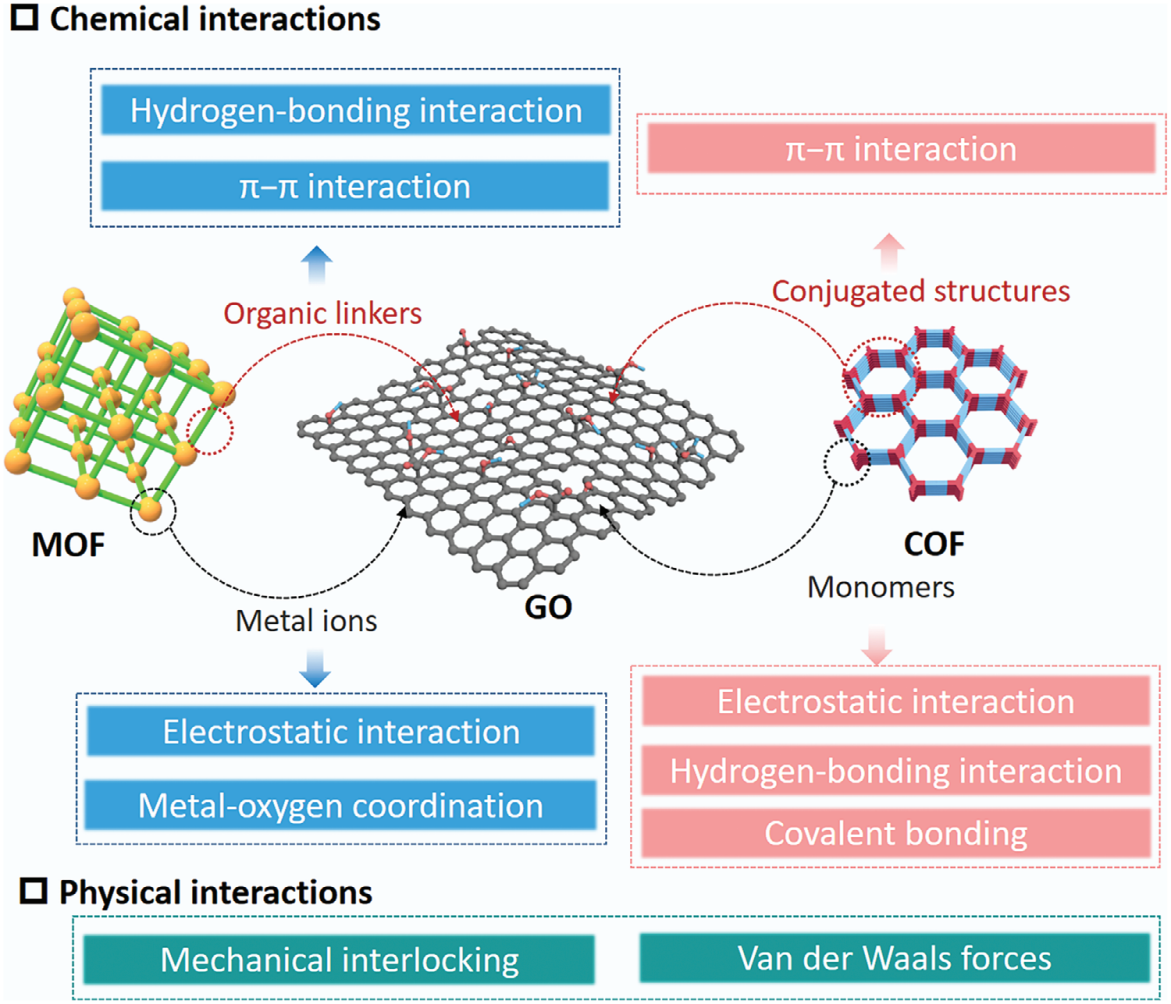
The chemical and physical interactions between MOFs/COFs and GO.

Understanding the chemical and physical interactions between MOFs/COFs and GO is crucial for designing and fabricating hybrid materials with enhanced functionality and performance in various applications (**Figure** **11**). Chemical interactions: 1) π–π interactions: MOFs/COFs and GO both possess aromatic ring structures capable of engaging in π–π stacking interactions.^[^ ^189^, ^190^ ^]^ This noncovalent interaction helps in the stable assembly of MOF or COF particles on the surface of GO sheets, facilitating composites with extensive interfacial contact crucial for effective electron and mass transfers, especially in catalysis and energy storage applications. 2) Hydrogen bonding: the oxygen‐containing functional groups (e.g., hydroxyl, epoxy, and carboxyl groups) present on the surface of GO can form hydrogen bonds with MOF or COF components.^[^ ^191^, ^192^ ^]^ For instance, in COFs, which are often composed of boronic acid or amine linkers, these groups can participate in hydrogen bonding with the oxygen groups on GO. This interaction aids in the dispersion of GO within the MOF/COF matrix and improves the structural integrity of the hybrid material. 3) Coordination bonding: in some cases, metal nodes in MOFs can directly coordinate to oxygen‐containing functional groups on GO.^[^ ^193^ ^]^ This strong chemical bonding contributes to the enhanced mechanical strength of the composite and ensures a robust interface for effective load transfer, which is particularly beneficial in mechanical reinforcement applications. 4) Electrostatic interactions: the negatively charged surface of GO can attract positively charged species, including metal ions or certain organics, facilitating their incorporation into the MOF/COF structure. This interaction can be utilized for the pre‐organization of MOF precursors or as a driving force for COF polymerization on the GO surface.^[^ ^190^ ^]^

Physical interactions: 1) Mechanical interlocking: at the macro‐scale, mechanical interlocking may occur when MOF/COF particles interdigitate with the layered structure of GO, enhancing the mechanical stability and strength of the composite. This is more relevant in 3D structured hybrids like aerogels or foams.^[^ ^124^ ^]^ 2) Van der Waals forces: although weaker than the aforementioned chemical interactions, van der Waals forces between MOF/COF components and GO contribute to the overall stability and cohesion of the composite material.

COFs generally have more intense chemical interactions with graphene/GO due to their covalent nature, forming stronger covalent bonds. In contrast, the interactions between MOFs and graphene/GO are relatively weak. As a result, COF–graphene/GO hybrids tend to be more stable due to these stronger covalent bonds, while MOF–graphene/GO hybrids may be less stable, especially in aqueous environments, due to the potential hydrolysis of metal–ligand bonds. The nature of these bonds also affects surface coverage. COFs can potentially achieve more uniform and complete surface coverage on graphene/GO due to their 2D nature and strong covalent bonds.^[^ ^105^ ^]^ On the other hand, MOFs may form more discrete particles on the graphene surface, leading to less uniform coverage.^[^ ^194^ ^]^ In terms of interactions, COFs may have more predictable and uniform interactions across their structure due to their covalent nature. However, MOFs can offer more selective interactions due to their diverse metal nodes and functional groups, providing greater versatility in certain applications. These forces, though nonspecific, can impact the assembly process and the final material's porosity and surface area. Therefore, a deep comprehension and strategic manipulation of these interactions can pave the way for innovative approaches in fabricating MOF/COF‐graphene aerogels and expand horizons in the realm of advanced materials.

#### 3.3.1 Postsynthetic Hybridization Strategy

**Figure 12:**
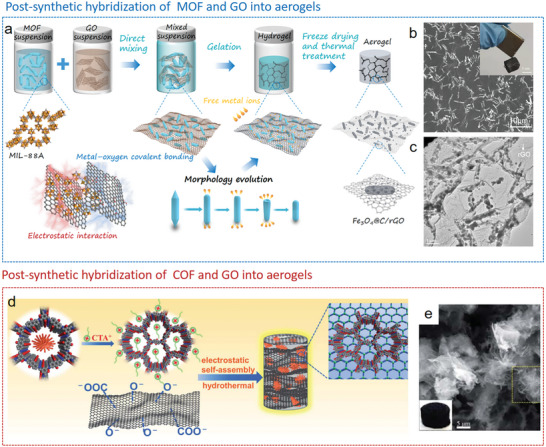
MOF/COF‐graphene aerogels prepared by postsynthetic hybridization strategy. a) Schematic representation of the preparation process and b) SEM image of MIL‐88A/graphene aerogel. c) SEM image of MIL‐88A/graphene derived Fe_3_O_4_@C/graphene aerogel. Reproduced with permission.^[^ ^194^ ^]^ d) Schematic representation of the fabrication process and e) SEM image of DAAQ‐COF/graphene aerogel. Copyright 2022, Springer Nature. Reproduced with permission.^[^ ^197^ ^]^ Copyright 2021, The Royal Society of Chemistry.

Owing to the presence of hydrophilic oxygenated groups, GO sheets are easily dispersible in numerous polar solvents, particularly in water. This property facilitates the assembly of GO sheets with MOF^[^ ^194^, ^195^, ^196^ ^]^ or COF^[^ ^197^ ^]^ particles in aqueous solutions to form 3D MOF/COF‐reduced GO (rGO) composite gels. These gels can be formed either through a chemical/hydrothermal reduction process^[^ ^198^, ^199^, ^200^, ^201^, ^202^, ^203^ ^]^ or via mechanical stirring.^[^ ^195^ ^]^ Subsequently, they can be transformed into 3D MOF/COF‐rGO composite aerogels by freeze‐drying. The gel formation hinges on metal–oxygen coordination bonds or electrostatic interactions between GO sheets and metal ions of MOF particles, as well as π–π interactions or hydrogen‐bonding interactions between GO sheets and organic linkers (Figure 11).^[^ ^182^ ^]^ Diverse presynthesized MOF crystals were mixed with GO aqueous solution by vigorous stirring, and hydrogels were formed without any reduction agents or cross linkers. However, even though MOFs can be integrated into graphene aerogels by this method, the formed bulk structure is relatively weak and can easily collapse.^[^ ^195^ ^]^ In our recent work,^[^ ^194^ ^]^ we developed a green and efficient synthetic method for crafting MIL‐88A/rGO hybrid aerogels. This process involves the direct formation of gels from GO in an aqueous dispersion, triggered by 1D MIL‐88A nanorods under moderate heat (**Figure** **12**a). The gelation process facilitated by MIL‐88A occurs in two stages. Initially, the interaction between free Fe^3+^ ions and the oxygen‐containing functional groups on the GO surface overcomes the electrostatic repulsive force between the GO nanosheets. Subsequently, the GO nanosheets undergo cross‐linking, where Fe^3+^ ions enhance the stacking of the sheets, leading to the formation of a three‐dimensional network. By varying the initial concentrations of the MOF suspensions, the composition of the resulting aerogels can be precisely controlled. Through our synthetic strategy, the MIL‐88A derived C@Fe_3_O_4_ nanorods can be uniformly distributed on the surface of graphene sheets after pyrolysis (Figure 12b,c).

As example for COF‐rGO aerogels, an anthraquinone‐containing COF (DAAQ‐COFs) was first modified by a cationic surfactant and then mixed with GO nanosheets. Due to the strong electrostatic attraction and π–π interactions, the DAAQ‐COF was wrapped by rGO nanosheets and embedded in the 3D rGO network after the hydrothermal process (Figure 12d,e). The obtained DAAQ‐COF‐graphene aerogels showed an improved electrochemical accessibility of anthraquinone moieties and thus exhibited high specific capacitance and cyclability as electrode material.^[^ ^197^ ^]^

**Figure 13:**
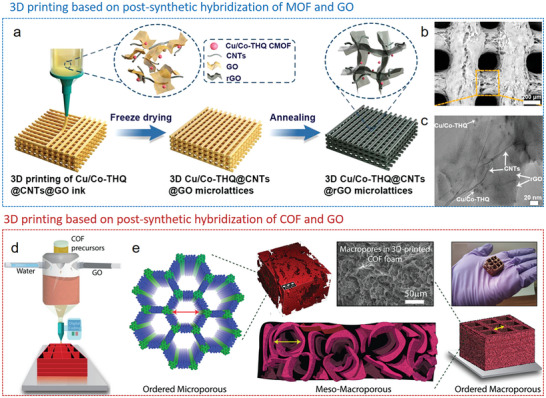
a) Schematic representation of the preparation process and b,c) microstructure of 3D Cu/Co‐THQ/rGO aerogel. Reproduced with permission.^[^ ^204^ ^]^ Copyright 2022, American Chemical Society. d) Schematic illustration of 3D printing and e) multiscale structures of COF‐GO foams. Reproduced with permission.^[^ ^205^ ^]^ Copyright 2020, American Chemical Society.

The shear‐thinning and viscoelastic response rheological behavior of MOFs or COFs mixed suspensions could be utilized to prepare inks for printing of 3D macro‐architecture via direct ink writing (DIW).^[^ ^204^, ^205^ ^]^ As an example, a series of 2D conductive MOF hybrid aerogels consisted of 2D MOF M‐tetrahydroxy‐1,4‐quinone (M‐THQ) (M = Cu, Cu/Co, or Cu/Ni), CNTs and GO sheets were achieved by DIW method and subsequent freeze‐drying process (**Figure** **13**a–c).^[^ ^204^ ^]^ In order to introduce ordered macropores into 2D COFs and engineer a 3D macro‐architecture into complex geometries, the 3D printing technique was also applied for COF‐GO foams.^[^ ^205^ ^]^ A COF precursor paste was firstly synthesized by mixing Tp and diamine in the presence of PTSA and water. Following the incorporation of a GO suspension, we obtained an appropriate ink characterized by shear‐thinning behavior and stable physicochemical properties, suitable for 3D printing (Figure 13d). Leveraging this approach, the multiscale structure of COF‐GO foams could be controlled (Figure 13e). The 3D‐printed COF‐GO foams exhibited consistent porosity, which included ordered macropores ranging from 1.5 mm to 2 cm, disordered macro and mesopores spanning 50 nm to 200 µm, and reticular micropores measuring between 2 and 2.2 nm, and it was demonstrated that such an architecture has potential for rapid and efficient uptake of organic and inorganic pollutants from water.

#### 3.3.2 In Situ Growth Strategy

The development of MOF/COF‐graphene aerogels through in situ growth can be categorized into two main approaches. The first involves the in situ cultivation of MOF/COFs directly on preexisting 3D graphene structures. The second is that MOF/COFs are synthesized on the surface of GO which is then assembled into 3D aerogels. Both approaches offer considerable advantages for the fabrication of MOF/COF‐graphene aerogels, allowing for controlled 3D porous architectures and uniform distribution of MOF/COF particles.

**Figure 14:**
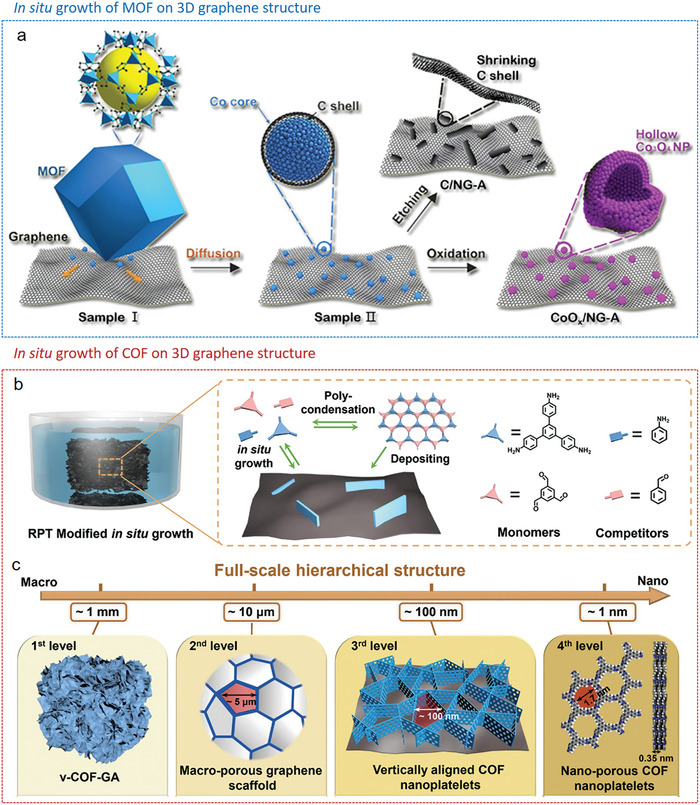
a) In situ growth of MOF on 3D graphene structures. Reproduced with permission.^[^ ^221^ ^]^ Copyright 2017, American Chemical Society. b) In situ growth of COF on 3D graphene structure, and c) the full‐scale hierarchical structure of COF/graphene aerogel. Reproduced with permission.^[^ ^131^ ^]^ Copyright 2022, Wiley‐VCH.

Various MOFs such as ZIF‐8,^[^ ^127^, ^206^, ^207^, ^208^, ^209^ ^]^ ZIF‐67,^[^ ^210^, ^211^, ^212^, ^213^ ^]^ MIL‐88‐Fe,^[^ ^127^, ^214^, ^215^ ^]^ MIL‐101‐Fe,^[^ ^216^ ^]^ Cu‐MOF,^[^ ^217^ ^]^ Cu‐BTC,^[^ ^218^, ^219^ ^]^ Mn‐MOF,^[^ ^220^ ^]^ Co‐MOF,^[^ ^221^, ^222^ ^]^ UiO‐66‐NH_2_^[^ ^223^ ^]^ have been incorporated with graphene by in situ growth of MOF nanocrystals on preformed 3D graphene hydrogels, aerogels or foams. As an example, MOFs (ZIF‐8 and or MIL‐88‐Fe) coated 3D graphene networks was developed by one‐pot solution immersion method.^[^ ^127^ ^]^ The acid‐treated 3D graphene networks were first immersed in the MOF precursors. After growing and vacuum drying, the 3D graphene/MOFs composites were obtained. The size of MOF nanocrystals could be tuned by the growth temperature and the concentration of MOF precursors. The desired 3D ZnO/graphene and Fe_2_O_3_/graphene composites, which were electrically conductive and possessed good mechanical properties, were then obtained by a thermal annealing process. A simplified approach was conducted by immersing graphene hydrogels in MOF precursor solutions. After reaction demonstrated by color changing, the Co‐MOF‐loaded graphene hydrogel was dried by supercritical CO_2_ drying. A post‐thermal treatment process was then applied to obtain Co oxide particles on graphene (**Figure** **14**a).^[^ ^221^ ^]^

Recently, vertically aligned 2D TAPB‐BTCA COF nanoplatelets were in situ grown on a graphene aerogel. As shown in Figure 14b, the 3D graphene aerogel was saturated with a reaction solution that included COF monomers (TAPB and BTCA), competitors (benzaldehyde and aniline) and a catalyst (Sc(OTf)_3_). The competitors regulate the reaction rate and mitigate the self‐coagulation of COF species, fostering a controlled synthesis environment. The resulting COF‐graphene aerogel displays a precisely defined hierarchical structure across various length scales (Figure 14c). On the macroscale, the graphene aerogel acts as a macroporous scaffold, strategically positioning vertically aligned COF nanoplatelets. These nanoparticles are designed to be arranged in a preestablished pattern at the micrometer scale. Each of these vertically aligned COF nanoplatelets, with an average thickness of about 20 nm and a lateral dimension of ≈200 nm, are uniformly distributed across the graphene sheets.^[^ ^131^ ^]^

To accomplish the in situ development of MOF/COF nanostructures on the GO surface, the simplest method involves initially blending MOF/COF precursors with a GO suspension at room temperature. This step is succeeded by heating the mixture under the solvothermal or reflux conditions required for the synthesis of MOF/COF. With this approach, GO sheets are considered more than just carbon nanomaterials; they are viewed as 2D macromolecules, embellished with numerous highly reactive oxygen‐containing functional groups.

**Figure 15:**
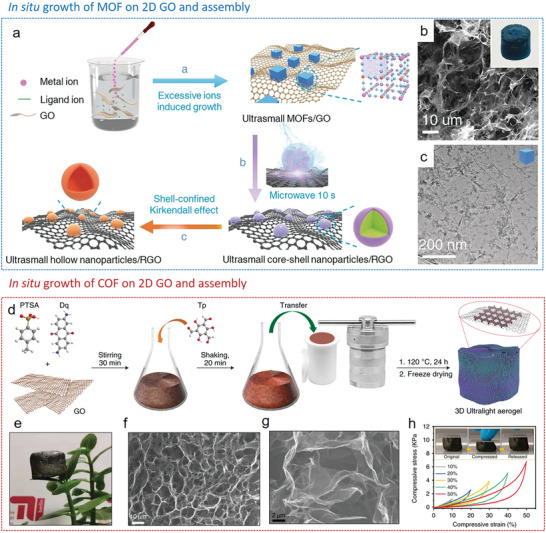
a) In situ growth of MOF on 2D GO and assembly, b) SEM and c) TEM images of MOF/graphene aerogel. Reproduced with permission.^[^ ^129^ ^]^ Copyright 2020, Wiley‐VCH. d) Fabrication of COF/graphene aerogel by in situ growth of COF on 2D GO and assembly strategy, e) the photograph, f,g) SEM images, and h) compressive stress–strain curves of COF/graphene aerogel. Reproduced with permission.^[^ ^105^ ^]^ Copyright 2020, Springer Nature.

A series of recent work demonstrated that Prussian Blue (PB) or Prussian Blue analogues (PBA)/graphene aerogels could be prepared by excessive metal ions induced self‐assembly strategy (**Figure** **15**a).^[^ ^128^, ^129^, ^224^, ^225^ ^]^ Specifically, when an excess of metal ions (Fe^3+^, Co^2+^, Ni^+^) was added to a homogenous solution of GO and cyanometallate ions ([Fe(CN)_6_]^4−^, [Co(CN)_6_]^3−^, [Ni(CN)_4_]^2−^), some of the metal ions reacted with cyanometallate anions to form PB/PBAs. Concurrently, the excess metal ions adsorbed onto the surface of the forming PB/PBAs. This process facilitates the deposition of PB/PBAs nanoparticles on the GO, which is enhanced by electrostatic and coordination interactions with GO's oxygen‐containing groups, while also preventing the excessive growth of PB/PBAs. After freeze‐drying and post‐treatment, nanostructured PB/PBAs‐graphene aerogels were obtained (Figure 15b,c), which were further applied in the field of electrochemical energy storage and conversion.

Attributed to the 2D structure and π‐conjugation of 2D COFs, they are well‐suited for forming composites with graphene.^[^ ^105^, ^130^ ^]^ In our recent work, a COF‐rGO hydrogel was synthesized through an in situ reaction involving the organic linkers Tp and Diaminoanthraquinone (Dq) in the presence of GO (Figure 15d). Due to the presence of oxygen‐containing functional groups on GO, Dq molecules interact and even become covalently grafted to the GO surface during the hydrothermal reaction. This interaction facilitates the uniform growth of a thin, continuous layer of TpDq‐COF layer along the surface of rGO nanosheets, with a thickness ranging from 2.9 to 6.0 nm. Following the freeze‐drying of the resulting hydrogel, an ultralight (7 mg cm^−3^), hierarchical porous COF‐rGO aerogel was formed (Figure 15e–g). This aerogel shows excellent conductivity, redox activity, and strong mechanical strength, enduring strains up to 50% without any compromise to its 3D structure. These characteristics contribute to its outstanding absorption and electrochemical properties (Figure 15h).^[^ ^105^ ^]^

### 3.4 MOF/COF‐MXene Hybrid Aerogels

MXenes, an emerging family of 2D transition metal carbides and nitrides, are being actively investigated as building blocks to construct well‐connected 3D porous architectures with other materials such as CNTs, graphene, polymer, or metal‐based particles attributed to their abundant surface functional groups (─OH, ─O, and ─F) and unique metallic conductivity.^[^ ^226^, ^227^, ^228^ ^]^

**Figure 16:**
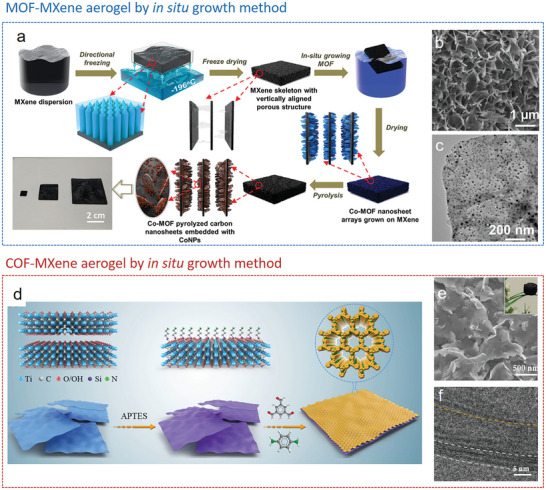
MOF‐MXene aerogel: a) schematic illustration of the fabrication process, b) SEM image and c) TEM image of the Co‐MOF‐MXene‐derived foam. Reproduced with permission.^[^ ^230^ ^]^ Copyright 2020, Wiley‐VCH. COF‐MXene aerogel: d) schematic illustration of the fabrication process and e) SEM image of MXene@COF scaffold. f) HRTEM image of MXene@COF nanosheets. Reproduced with permission.^[^ ^232^ ^]^ Copyright 2021, Wiley‐VCH.

Recently, MOF‐MXene^[^ ^229^, ^230^, ^231^ ^]^ and COF‐MXene^[^ ^232^ ^]^ aerogels have been fabricated by the in situ growth method. For example, a Co‐MOF‐MXene foam was fabricated by growing the ZIF‐L directly onto the surface of a vertically aligned porous Ti_3_C_2_T_x_ MXene skeleton.^[^ ^230^ ^]^ Cobalt nanoparticle–carbonaceous nanosheets–MXene foams can be obtained by a subsequent pyrolysis process and used for efficient and stable solar‐water desalination with good salt resistance (**Figure** **16**a–c). In another approach, ZIF‐67‐MXene aerogel was prepared using the metal ions as both crosslinkers to coordinate with the oxygen‐containing functional groups of MXene to form a hydrogel and nucleation sites for the in situ growth of ZIF‐67 particles. After carbonization and sulfidation processes, the final aerogel showed promising properties as electrode materials for alkali‐ion batteries.^[^ ^229^ ^]^

COF‐MXene aerogels were assembled using aminopropyltriethoxysilane modified MXene combined with organic linkers, 1,3,5‐triformylbenzene (TFB), and *p*‐phenylenediamine (PDA) in 1,4‐dioxane. COF‐LZU1 could subsequently be cultivated on the MXene nanosheets through Schiff base condensation, which occurs between the aldehydes and amino groups of the respective monomers and the MXene, facilitated by an acetic acid catalyst (Figure 16d). The resulting 3D scaffolds facilitate rapid charging in lithium metal batteries by hosting lithium (Figure 16e,f).^[^ ^232^ ^]^

COF‐based aerogels, including COF/graphene, COF/(bio)polymer, and COF/MXene composites, have emerged in the past five years, garnering significant attention due to their unique properties and potential applications. **Table** **3** outlines the differences in synthesis methods, amorphous nature, properties, and applications of each type of COF‐based aerogels.

**Table 3:** Monomers, synthesis method, and applications of typical COF‐based aerogels.

COF‐based aerogels	COF	Monomers	Synthesis method	Crystallinity	Loading content [wt%]	Density [mg cm^−3^]	SSA (m^2^/g)	Properties	Applications	Refs.
COF/chitosan	COF‐IL	1,3,5‐triformylphloroglucinol (Tp); 2‐methyl‐1,4 benzenedicarboxylic acid	Postsynthetic hybridization	High crystallinity	80	32–35	103.3	High mechanical strength	CO_2_ cycloaddition reaction	[144]
COF/chitosan	TpPa‐SO_3_H	Tp, 2,5‐diaminobenzenesulfonic acid (Pa‐SO_3_H)	Postsynthetic hybridization	High crystallinity	75	–	30.03	Compressible (5 cycles)	Sulfamerazine removal	[157]
COF/cellulose	Tp‐Pa‐2SO_3_H	Tp, 2, 5‐diamino‐1, 4‐benzenesulfonic acid (Pa‐2SO_3_H)	Postsynthetic hybridization	High crystallinity	–	–	–	–	Water Harvesting, Power Generation	[151]
COF/cellulose	AZO‐3	Tp, 4,4′‐diaminoazobenzene (AZO‐NH_2_)	Postsynthetic hybridization	High crystallinity		4.5–27.5	188.27	Elasticity (500 cycles)	Uranium extraction	[233]
COF/cellulose	COF‐LZU1	1,3,5‐Triformylbenzene (Tb), *p*‐phenylenediamine (Pa)	Postsynthetic hybridization	High crystallinity	50	15	–	–	Iodine Uptake	[234]
COF/PVA	DPP‐TPA COF	5,5′‐(2,5‐Bis(2‐ethylhexyl)‐3,6‐dioxo‐2,3,5,6‐tetrahydropyrrolo[3,4‐c]pyrrole‐1,4‐diyl)bis(thiophene‐2‐carbaldehyde) (DPP‐CHO), triaminotriphenylamine (TPA‐NH_2_)	Postsynthetic hybridization	High crystallinity	8.6	–	–	Hydrophilicity, low thermal conductivity	Solar steam generation	[150]
COF/graphene	Tp‐Dq	Tp, diaminoanthraquinone (Dq)	In situ growth and assembly	High crystallinity	–	7	246	Elasticity, electrically conductive	organic solvent adsorption, supercapacitors	[105]
COF/graphene	COF‐SO_3_H	Tp, 2,5‐diaminobenzenesulfonic acid (DASA)	In situ growth and assembly	Low crystallinity	–	–	–	Tunable wettability, light‐absorbing	Solar‐driven water generation	[130]
COF/graphene	Tp‐DB‐SO_3_Na	Tp, sodium 2,5‐diaminobenzenesulfonate (DB‐SO_3_Na)	In situ growth and assembly	Low crystallinity	–	7.1–10.6	149–185	–	Dye adsorption	[235]
COF/graphene	Bpy‐COF‐Co	1,3,5 triformylbenzene (TFB), 2,2′‐bipyridine‐5,5′‐diamine (Bpy)	In situ growth on 3D framework	High crystallinity	–	–	412	Electrically conductive, rich accessible active sites	Overall water splitting	[236]
COF/graphene	TAPB‐BTCA	1,3,5‐tris(4‐aminophenyl)‐benzene (TAPB), 1,3,5‐benzenetricarbaldehyde (BTCA)	In situ growth on 3D framework	High crystallinity	57.8	–	282	Electrically conductive, rich accessible active sites	Supercapacitor	[131]
COF/MXene	COF‐LZU1	TFB, p‐phenylenediamine (PDA)	In situ growth and assembly	Low crystallinity	–	–	239	Electrically conductive	Lithium metal batteries	[232]

COF/(bio)polymer aerogels are typically prepared through a postsynthetic hybridization strategy. This involves mixing COF powders with (bio)polymers (e.g., cellulose, chitosan, and PVA) in solution, followed by gelation and freeze‐drying. This method can achieve relatively high COF loading content (up to 80 wt%), which can be adjusted by varying the ratio of COF powders to (bio)polymer. These aerogels are often used in environmental remediation applications.

COF/graphene aerogels are typically synthesized through in‐situ growth of COFs on 2D GO sheets or 3D graphene frameworks. The loading content or thickness of COF layers can be adjusted by varying the concentration of COF monomers. These aerogels exhibit higher surface area and porosity, better mechanical strength and flexibility. Furthermore, COF/graphene aerogels provide accessible active sites and efficient transport pathways for both ions and electrons, making them beneficial for energy storage applications (e.g., supercapacitors and batteries) and catalysis.

The COF‐based aerogel family shows great promise for researchers. In the future, there is significant potential for exploring novel properties, improving performance, and discovering cutting‐edge applications in this field. As this area of study continues to evolve, it is likely to attract increased attention from scientists and engineers across various disciplines, leading to innovative breakthroughs and expanded utilization of these versatile materials.

## 4 Emerging Applications

By harnessing the intrinsic microporosity of MOFs/COFs and integrating it with the 3D meso‐ and macroporosity of aerogels, MOF/COF‐based aerogels are expected to exhibit superior accessibility to active sites compared to their pure MOF/COF powder counterparts. Their hierarchical pore configuration, which facilitates the transportation of ions, electrons, gases, and liquids, is acknowledged as a crucial architectural attribute for their real‐world utility. This section provides an overview of various potential uses for 3D MOF/COF‐based macrostructures, including applications in environmental remediation, gas uptake and separation, water harvesting, energy storage, and catalysis.

### 4.1 Environmental Remediation

**Figure 17:**
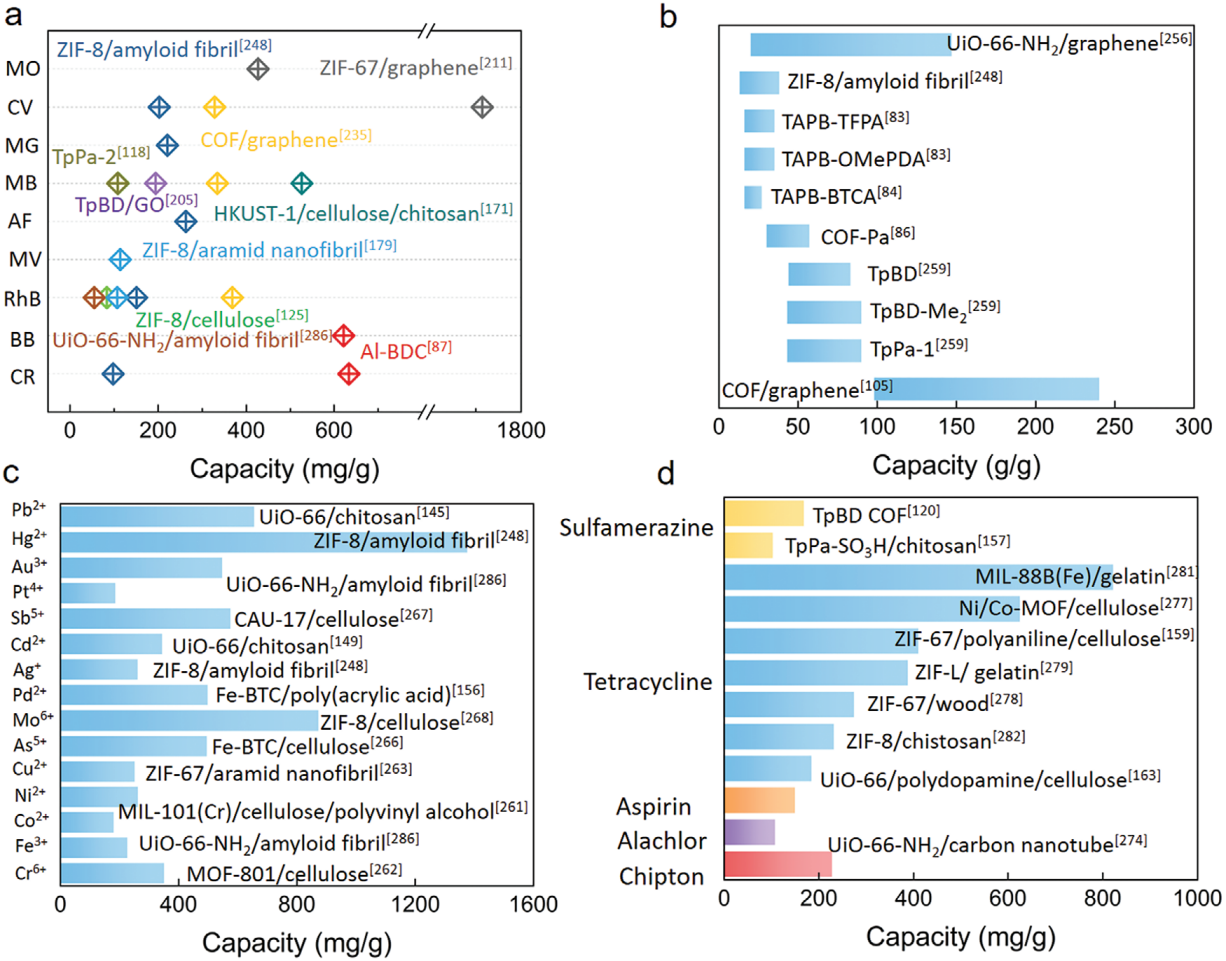
Typical MOF/COF‐based aerogels for contaminant removal from water. a) Dyes, b) organic solvents, c) metal ions, and d) pesticides and pharmaceuticals.

MOFs/COFs have shown notable capabilities in the filtration and detoxification of pollutants from aquatic and atmospheric environments.^[^ ^237^, ^238^ ^]^ Despite this potential, their widespread practical deployment is hindered by challenges associated with their powdery form, including the blockage of conduits, complications in material retrieval, and possible ecological risks due to their inherent delicacy. To address these issues, research has pivoted toward crafting macroscale structures derived from MOFs and COFs to surmount these obstacles.^[^ ^239^, ^240^, ^241^, ^242^ ^]^ Macroscopic MOF/COF‐based aerogels, in particular, are undergoing extensive evaluation for their efficacy in purging a spectrum of contaminants ranging from dyes, oils, and organic solvents to heavy metals, radionuclides, pesticides, and antibiotics in water, as well as particulate pollutants in the air (**Figure** **17**). Their vast specific surface area, adaptable surface chemistry, and improved mechanical strength make them highly effective for such tasks.^[^ ^243^, ^244^, ^245^, ^246^, ^247^, ^248^ ^]^ Moreover, the photothermal and photocatalytic properties evident in many MOF/COF materials are paving the way for innovative uses like photocatalytic degradation of pollutants and solar‐powered water purification technologies.^[^ ^249^, ^250^, ^251^, ^252^ ^]^

#### 4.1.1 Adsorption of Dyes

The release of organic dyes from textile manufacturing processes can severely harm aquatic ecosystems. MOF/COF‐based aerogels have been shown to effectively remove a diverse range of dyes from water (Figure 17a). This is achieved through adsorption, a process facilitated by the aerogels' intricate hierarchical porous architectures and the electrostatic interactions between the dyes and the material.^[^ ^249^, ^253^, ^254^ ^]^ Al‐BDC aerogel processes high adsorbing capacities of 633.4 mg g^−1^ for Congo Red (CR) and 621.3 mg g^−1^ for Brilliant Blue R‐250 (BB). This performance can be credited to the presence of additional mesopores in the MOF aerogels, which are readily accessed by organic dye molecules.^[^ ^87^ ^]^ Beyond the utilization of pure MOF aerogels, embedding MOF particles in substance matrices such as biopolymers or graphene aerogels has proven to be an effective method for introducing microporosity and enhancing mechanical strength. For instance, fibrous cellulose/ZIF‐8 aerogels, which are both flexible and sturdy, possess an adsorption rate of 0.036 g mg^−1^ h^−1^ and equilibrium adsorption capacity of 83.3mg g^−1^ for Rhodamine B (RhB), which surpasses that of pure ZIF‐8 powders.^[^ ^125^ ^]^ Furthermore, MOF/graphene aerogels have been crafted for the dual removal of cationic and anionic dyes from water systems. Concurrently, reduced graphene oxide targets cationic dyes through a synergistic blend of electrostatic forces, π–π stacking interactions, and hydrogen bonds. Illustratively, a ZIF‐8/graphene aerogel has demonstrated adsorption capacities reaching 1714.2 mg g^−1^ for Crystal Violet (CV) and 426.3 mg g^−1^ for Methyl Orange (MO), showcasing its efficacy in dye removal applications.^[^ ^211^ ^]^

**Figure 18:**
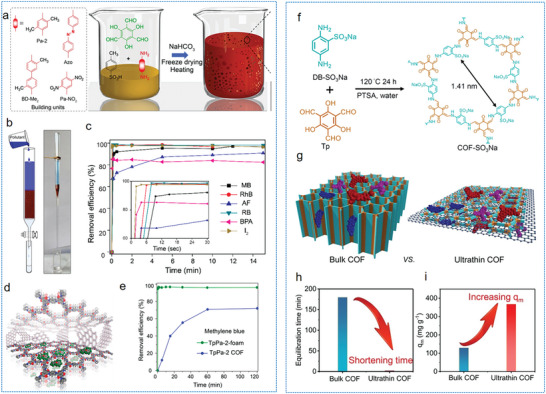
a) Schematic representation of COF foam synthesis by an in situ gas‐foaming technique. b,c) Fast removal of pollutants from water using COF foam. d) The model about how dye molecules are situated within the micropores of the COF foam. e) The comparsion of time‐dependent removal efficiency of COF‐foam and pristine COF. Reproduced with permission.^[^ ^118^ ^]^ Copyright 2019, American Chemical Society. f) Formation and pore structure of COF‐SO_3_Na. g) An illustration compares the dye adsorption mechanism on bulk COF with that on ultrathin COF. h,i) The equilibrium time and maximum Rhodamine B adsorption capacities of bulk COF and ultrathin COF. Reproduced with permission.^[^ ^235^ ^]^ Copyright 2022, Wiley‐VCH.

COF foams derived from 2D COF networks are also promising candidates for pollutant removal. The efficacy of the foams is due to the substantial surface area provided by the 2D COF lattice, coupled with the hierarchical macropores present within the 3D structure. These features collectively facilitate the effective capture and elimination of pollutants. COF foams, prepared by an in situ gas‐phase foaming technique, demonstrated the capability to rapidly segregate various dyes (**Figure** **18**a), including RhB, Methylene Blue (MB), and Acid Fuchsin (AF), from water. These foams achieved a removal efficiency greater than 95% within just 10 s (Figure 18b,c). Notably, the TpPa‐2‐foam could remove over 99% of MB in the same duration, outperforming pure COF powder which only managed to efficiently remove 70% after 2 h (Figure 18d,e).^[^ ^118^ ^]^ Additionally, 3D‐printed COF‐GO foams feature a high interconnected macroporous volume (55%) that enhances water flow (1.13 × 10^−3^ m s^−1^), further facilitating mass transport and adsorption.^[^ ^205^ ^]^

The design of adsorbents for the removal of organic dyes needs to consider both, the size of adsorbent pores and the dye molecules, i.e., whether the inside of the pores is accessible for the pollutant molecules. However, the micropore/small mesopores (mostly 1–4 nm) and the highly stacked nature of COFs can hinder the pollutants from entering the pores, especially for larger dye molecule. Recently, we investigated the impact of COF layer thickness on the efficiency of organic dye removal by using graphene as a surface template to develop ultrathin sulfonate anionic COF/graphene aerogels (Figure 18f).^[^ ^235^ ^]^ We found that ultrathin COFs with a layer thickness of ≈2 nm showed highest adsorption performance owing to the optimized active site exposure and electrostatic attraction (Figure 18g). Moreover, ultrathin COF exhibited much faster removal and higher uptake capacities for various organic pollutants regardless of their sizes compared with bulk COF powder. For example, ultrathin COF could capture >99% of RhB from water within 3 min, while it took 3 h for the bulk COF powder (Figure 18h). Meanwhile, the maximum uptake capacity improved near three times when using ultrathin COF as adsorbent (Figure 18i). Therefore, the ultrathin COF layer with large amount of adsorption sites combined with the macropore channels of graphene network confirmed that efficient and fast wastewater treatment is possible with such aerogels.

The repeatability of MOF/COF aerogels for dye adsorption primarily depends on their structural stability and regeneration capability. Many MOF/COF aerogels can be regenerated through washing or solvent treatment, enabling multiple uses. For instance, the TpPa‐2‐foam, COF‐GO foam, and ultrathin COF have demonstrated excellent reusability, maintaining their performance over five consecutive cycles. Between each cycle, these COFs were treated with acetone to recover the adsorbed pollutants. This straightforward regeneration process allowed for immediate reuse in subsequent cycles.

Furthermore, the stability of MOFs/COFs in water is noteworthy, as it prevents the release of adsorbent particles, thus eliminating the risk of secondary pollution in the treated water. This characteristic is crucial for maintaining water quality throughout the treatment process. The ability to regenerate and reuse these aerogels not only enhances their economic viability but also contributes to sustainable water treatment practices. As research in this field progresses, the development of even more stable and easily regenerable MOF/COF aerogels could lead to more efficient and cost‐effective water purification technologies.

#### 4.1.2 Removal of Organic Solvents and Oils

Oil spills and releases of organic hydrocarbon‐based solvents pose significant threats to both marine and freshwater ecosystems. For effective remediation, it is crucial to employ lightweight sorbents characterized by a high specific surface area and a strong affinity for oil. MOF/COF‐based aerogels exhibit considerable removal capacity for organic solvents as well as oils (Figure 17b).^[^ ^86^, ^105^, ^255^, ^256^, ^257^, ^258^, ^259^ ^]^

Polystyrene/ZIF‐8 foams can be installed in various large‐scale filtration units for continuous separation, achieving successful separation of all tested emulsions with oil purities ranging from 99.2 to 99.7 wt%. These foams operate at high fluxes, with rates between 3500 and 4400 L m^−2^ h^−1^ bar^−1^, thus much higher than commercial filtration membranes, suggesting the potential for industrial applications.^[^ ^260^ ^]^ In a recent work, imine‐based COF aerogels prepared by the sol‐gel method exhibit good absorption capacity (32 g g^−1^) of toluene and good recyclability.^[^ ^82^ ^]^ The marriage of 2D COFs and graphene aerogels further improve its mechanical property, and the absorption capacity of oil and organic solvent can reach to 240 g g^−1^.^[^ ^105^ ^]^

#### 4.1.3 Removal of Heavy Metals and Radionuclides

The occurrence of heavy metals in aquatic ecosystems is alarming due to their significant toxicity to both human health and environmental systems. The hierarchical porous structures and abundant active sites render MOF/COF‐based aerogels ideal for removing heavy metal ions including Co^2+^,^[^ ^261^ ^]^ Ni^2+^,^[^ ^261^ ^]^ Cr^6+^,^[^ ^124^, ^262^ ^]^ Cu^2+^,^[^ ^263^ ^]^ Pb^2+^,^[^ ^145^, ^149^, ^264^, ^265^ ^]^ Cd^2+^,^[^ ^149^, ^176^, ^199^ ^]^ Pd,^[^ ^156^ ^]^ As^5+^,^[^ ^266^ ^]^ Sb^5+^,^[^ ^267^ ^]^ Hg^2+^,^[^ ^155^ ^]^ Mo^6+^,^[^ ^268^ ^]^ and radionuclides U(VI)^[^ ^212^, ^213^, ^269^, ^270^, ^271^, ^272^ ^]^ from water (Figure 17c). For example, flexible UiO‐66/cellulose aerogels with high UiO‐66 loading showed promising adsorption capacity of Cr^6+^. This effectiveness is credited to the aerogels' macroporous structure which facilitates easy access, and the capillary action within the mesopores that allows for rapid water absorption and efficient contact between the MOF micropores and the contaminants.^[^ ^124^ ^]^ Graphene aerogels have been shown to be effective adsorbents for the removal of heavy metal ions from water, thanks to their ion‐chelating properties and negatively charged functional groups. An improved adsorption performance of Cd^2+^ and Pb^2+^ (101.1 and 281.5 mg g^−1^) could be achieved after the uniform decoration of ZIF‐8 nanoparticles on graphene aerogels due to the extra binding sites.^[^ ^199^ ^]^

The macroscopic shape of MOF/COF aerogels, in contrast to the fine powders as COFs and MOFs are usually obtained, make them practical for real applications requiring continuous flow. Fe‐BTC‐polymer beads prepared by shaping co‐gelated hydrogels, showed performance in extraction of Pb^2+^ and Pd^2+^ from liquid streams. It was discovered that 1 g of the beads can purify more than 10 L of freshwater contaminated with highly toxic levels of Pb^2+^ (600 ppb) under continuous flow conditions. Furthermore, the beads demonstrated a high uptake capacity for Pd, reaching 498 mg g^−1^, and were able to concentrate 7.8 wt% of Pd efficiently within the bead structure under continuous flow conditions.^[^ ^156^ ^]^ Solar‐driven water generation technology could also be used to purify wastewater containing mixed heavy metals. This approach focuses on reducing the concentration of heavy metal ions, ensuring cleaner water output. The concentration of heavy metal ions, such as Cu^2+^, Hg^2+^, Cd^2+^, Ag^+^, Cr^3+^, Ni^2+^, Ga^3+^, drops to 0.001 mg L^−1^ after purification using COF‐based solar generators, meeting the WHO standards of drinking water.^[^ ^130^ ^]^

The advent of the nuclear industry has given rise to radioactive pollution, which presents considerable risks to both human health and the environment. In light of this, the advancement of highly effective adsorbents tailored for uranium extraction is an urgent priority for exploration and implementation. A novel ternary MOF‐based aerogel composed of graphene, carbon nitride quantum dots, and ZIF‐67 has been utilized for the efficient extraction of uranium (VI) from contaminated sources. The notable adsorption capacity of this aerogel, amounting to 260.59 mg g^−1^, is primarily facilitated by the electrostatic attraction between the negatively charged MOF‐based aerogel structure and the uranium ions. The material exhibits rapid adsorption kinetics, achieving over 86% removal of uranium within just 75 min.^[^ ^213^ ^]^ Additionally, the uranium sequestration capabilities of these MOF‐based aerogel adsorbents can be further enhanced by leveraging photocatalysis to convert soluble uranium (VI) into its insoluble oxide form, uranium (IV).^[^ ^269^ ^]^

#### 4.1.4 Removal of Pesticides and Pharmaceuticals

Pesticides used in agriculture and antibiotics used in livestock farming are two categories of agro‐related organic contaminants that have become a significant concern. They are particularly worrying because many of these substances are persistent, posing substantial threats to both ecosystems and human health. ^[^ ^273^ ^]^ Notably, hydrophilic and compressible aerogels based on UiO‐66‐NH_2_ anchored to carbon nanotubes demonstrate promising efficiency in adsorbing herbicides such as chipton and alachlor.^[^ ^274^ ^]^ Additionally, MOFs/COFs‐based aerogels possess remarkable capacity for the adsorption of various active pharmaceutical compounds (Figure 17d), including ciprofloxacin,^[^ ^275^ ^]^ ibuprofen,^[^ ^276^ ^]^ naproxen,^[^ ^276^ ^]^ aspirin,^[^ ^163^ ^]^ sulfamerazine,^[^ ^120^, ^157^ ^]^ and tetracycline hydrochloride.^[^ ^159^, ^163^, ^277^, ^278^, ^279^, ^280^, ^281^, ^282^ ^]^

#### 4.1.5 Removal of Particulate Matter

MOF‐based aerogels were used to remove particulate matter from air, using a process similar to the removal of contaminants from water.^[^ ^173^, ^208^, ^283^, ^284^ ^]^ For example, ZIF‐8 nanoparticles anchored in graphene aerogels were effective for capturing particulate matter (PM) under both ambient and harsh environments. The 3D networks with tortuous and discontinuous macrochannels are capable of trapping PM particles, which further cover the MOF surface through electrostatic interaction between metal sites and PM particles. The capture efficiencies for PM2.5 and PM10 exceed 98.8% and 99.1%, respectively, at 200 °C with a flow rate of 30 L min^−1^.^[^ ^208^ ^]^

### 4.2 Gas Uptake and Separation

The substantial surface area and extensive pore volume of MOF and COF aerogels enhance the adsorption and separation of gas molecules, including CO_2_,^[^ ^87^, ^91^, ^206^, ^285^, ^286^, ^287^, ^288^, ^289^, ^290^, ^291^, ^292^, ^293^, ^294^, ^295^ ^]^ energy‐related gases (H_2_, CH_4_ and C_2_H_2_),^[^ ^87^, ^91^, ^289^, ^296^ ^]^ and vapors (hexanal, hexane, toluene, *p*‐xylene, benzene, methanol, and iodine).^[^ ^83^, ^84^, ^87^, ^100^, ^104^, ^234^, ^297^, ^298^, ^299^ ^]^ As an example, Al‐BTC aerogels demonstrate a significantly enhanced CO_2_ uptake at high pressure, achieving 627 mg g^−1^ at 298 K and 50 bar. This is primarily due to the robust physisorption of CO_2_ onto the unsaturated metal sites located on the surfaces of the intercrystallite mesopores.^[^ ^87^ ^]^ The absolute CH_4_ uptake values of meso/microporous HKUST‐1 and UiO‐66 monoliths can reach 309 and 301 cm^3^ (STP) cm^−3^ (298 K, 100 bar).^[^ ^91^, ^300^ ^]^ A water‐stable and hydrophobic CAU‐3 aerogel, characterized by a porous network of cubic nanoparticles (≈50 nm), exhibits high efficiency in capturing hexanal under wet conditions. Additionally, these aerogels can be easily applied to nonwoven fibers using a straightforward dip‐coating technique, demonstrating both high hexanal removal efficiency and excellent regenerability at low temperatures.^[^ ^104^ ^]^

**Figure 19:**
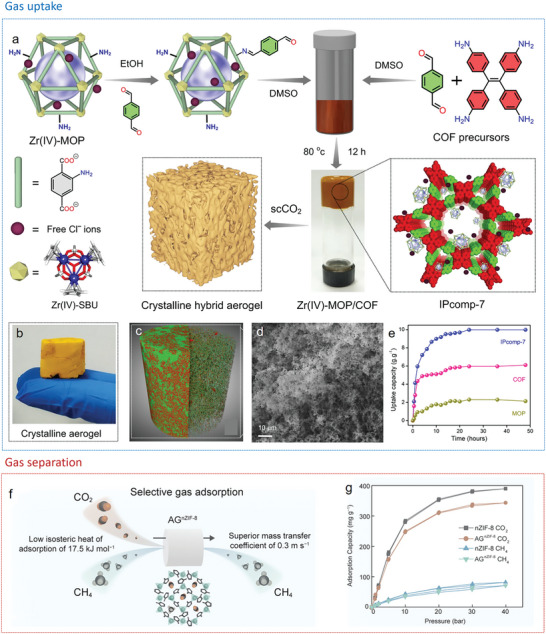
a) Schematic representation of fabrication procedure, b) photograph, c) 3D X‐ray tomographic image, d) SEM image, and e) gaseous iodine uptake performance of NH_2_‐Zr(IV)‐MOP embedded COF aerogel (IPcomp‐7). Reproduced with permission.^[^ ^298^ ^]^ Copyright 2024, Springer Nature. f) Selective gas adsorption performance of nZIF‐8‐CNF aerogel. e) Gravimetric equilibrium adsorption isotherms of CO_2_ and CH_4_ in pristine nZIF‐8 powder and aerogel. Reproduced with permission.^[^ ^122^ ^]^ Copyright 2022, Wiley‐VCH.

In the case of TAPB‐BDCA COF aerogels, the hierarchically interconnected pore structures are favorable to iodine vapor transport, and the iodine uptake capacity can reach 8.15 g iodine/1.0 g COF aerogel, again superior to pure COF powders. Furthermore, the iodine adsorption performance of the COF aerogel under conditions relevant to industrial applications significantly surpassed that of traditional adsorbents.^[^ ^84^ ^]^ Recently, Fajal et al reported the synthesis of a cationic metal–organic polyhedra (MOP) and imine COF hybrid aerogel, named IPcomp‐7. As shown in **Figure** **19**a, the process began with the modification of an amino‐functionalized MOP using terephthaldehyde to add functional groups capable of subsequent reaction with COF precursors, specifically tetraamino‐tetraphenylethylene, along with an additional quantity of terephthaldehyde, thus generating the hybrid wet‐gel materials. After undergoing reactivation and solvent‐exchange processes, this wet‐gel was transformed into an aerogel using a supercritical CO_2_ drying method, culminating in the production of a lightweight, hierarchically porous Zr(IV)‐MOP/COF aerogel (Figure 19b–d). The IPcomp‐7 hybrid aerogel demonstrated a remarkable maximum adsorption capacity of vapor‐phase iodine, reaching 9.98 g g^−1^ within a 24 h operational period. This performance substantially exceeds that of the pristine MOP, which is 2.31 g g^−1^, and the COF aerogel, which is 6.11 g g^−1^ (Figure 19e).^[^ ^298^ ^]^

In comparison to pure MOF aerogels, nZIF‐8/CNF hybrid aerogels prepared by freeze‐casting showed a better performance in gas separation of CH_4_ and CO_2_ (Figure 19f). The uptake capacities of CO_2_ and CH_4_ are 343 and 71 mg g^−1^, respectively (Figure 19g). In a 50/50 (mol/mol) gas mixture relevant to biogas upgrading, the nZIF‐8/CNF aerogel displays a selectivity of 8:1 (CO_2_:CH_4_) at equilibrium. The material demonstrates stable and reversible CO_2_ uptake through cyclic low‐pressure adsorption‐desorption tests, featuring efficient regeneration via simple pressure reduction. Additionally, the separation of C_2_H_2_/CO_2_ was explored using a moldable and processable NKCOF‐12 foam. Owing to its uniform ultramicroporous channels (0.58 nm) and enriched binding sites, the NKCOF‐12 foam delivers outstanding C_2_H_2_/CO_2_ separation performance, achieving a higher C_2_H_2_ purity (99.3%) compared to benchmark materials

### 4.3 Sustainable Energy‐Water Technology

#### 4.3.1 Solar‐Driven Water Evaporation

Solar‐driven water evaporation technology has been considered as one of most promising methods to mitigate water shortage.^[^ ^301^, ^302^ ^]^ The generation rate of clean water strongly depends on the evaporators, which are designed to optimize water transport and thermal management. COFs usually possess low thermal conductivity, a tunable bandgap, high surface areas, and defined functional groups at the surface, thus are suitable components for evaporators.^[^ ^303^, ^304^ ^]^ COF‐based aerogel evaporators have been developed very recently.^[^ ^85^, ^130^ ^]^ A diketopyrrolopyrrole (DPP)‐based 2D COF supported by 3D PVA scaffold has been designed and fabricated toward solar steam generation. The COF powder is directly mixed with PVA solution to obtain a hybrid hydrogel under the activation with hydrochloric acid. The hybrid hydrogel shows good light absorption, low thermal conductivities and good water transport ability. Thus, a steam generation rate of 2.5 kg m^−2^ h^−2^ with an energy efficiency of 93.2% has been achieved under one sun irradiation. Moreover, the COF/PVA hydrogel exhibits good performance for seawater desalination, as the collected purified water meets drinkable water standards.^[^ ^150^ ^]^

**Figure 20:**
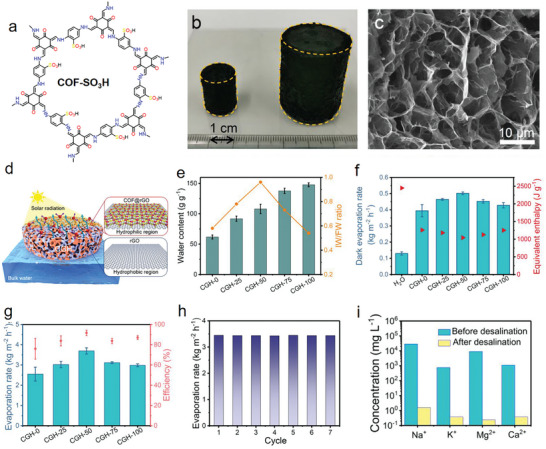
a) The structure of COF‐SO_3_H. b) Photographs and SEM image of COF/graphene aerogel. d) Scheme of the COF/graphene CGH evaporator. e) Water content, and the proportion of intermediate water (IW) and free water (FW) in CGHs. f) Comparison of dark evaporation rate and evaporation enthalpy between pure water and water in CGHs. g) Water evaporation rate from CGHs and energy efficiency under 1 sun irradiation. h) Stability test using simulated seawater with CGH‐50 across seven cycles. i) Ion concentration of simulated seawater before and after desalination. Reproduced with permission.^[^ ^130^ ^]^ Copyright 2022, American Chemical Society.

Despite the development of various photothermal materials for solar water generation, the evaporation rates of most existing materials remain suboptimal. Therefore, designing innovative materials to boost the rate of clean water production is crucial. Our recently developed COF/graphene dual‐region hydrogel (CGH) evaporator, crafted through a straightforward in situ growth strategy, incorporates both hydrophilic COF and hydrophobic graphene regions within a single material. This design aims to enhance the efficiency of solar water evaporation. The hydrophilic, sulfonic acid‐functionalized COF (COF‐SO_3_H) was just partially deposited onto hydrophobic rGO surface, forming hydrophilic and hydrophobic regions (**Figure** **20**a–d). Through accurate adjustment of both wetting regions, the hybrid hydrogel exhibited effective light‐harvesting, high water transport rate, tunable wettability (water contact angles from 69.7 ± 1.7° to 130.6 ± 4.6°), optimized water content (from 61.8 to 147.6 g g^−1^) and water state (Figure 20e), as well as lowered energy demand for water vaporization (1043 J g^−1^ for CGH‐50 vs 2450 J g^−1^ for bulk water, Figure 20f). Serving as a solar absorber, the optimized CGH‐50 demonstrated a high vapor generation rate of 3.69 kg m^−2^ h^−1^ with an impressive 92% energy efficiency under 1 sun irradiation (Figure 20g). Moreover, the CGH‐50 sample achieved an evaporation rate of 3.45 kg m^−2^ h^−1^ using simulated seawater under 1 sun illumination and maintained the high seawater evaporation performance for seven cycles (Figure 20h). The ion concentrations (Na+, K+, Ca^2+^, and Mg^2+^) in the collected water were significantly reduced by four orders of magnitude, and the desalination efficiency was estimated to exceed 99.98%. This performance meets the standards for drinking water (Figure 20i).^[^ ^130^ ^]^

In addition to seawater desalination, solar‐driven water evaporation technology has also been employed for the wastewater purification. Different from adsorptive purification, the solar vapor generation collects the evaporated water from the evaporator. This process can be in principle infinitely carried out because dyes are small molecules (usually a few nanometers in size) and would not block the water transport channels of evaporators (usually a few micrometers to tens micrometers, even hundreds micormeters). The COF/graphene dual‐region hydrogel we developed as solar evaporator was used to collect water from industrial dye wastewater with a purification efficiency of 100%, while the adsorption efficiency was only 87.5% under the same initial dye concentration. Notably, the evaporation rate of the hydrogel could still maintain 95% after four consecutive days and the water collected was clear and transparent.^[^ ^130^ ^]^

#### 4.3.2 Atmospheric Water Harvesting

**Figure 21:**
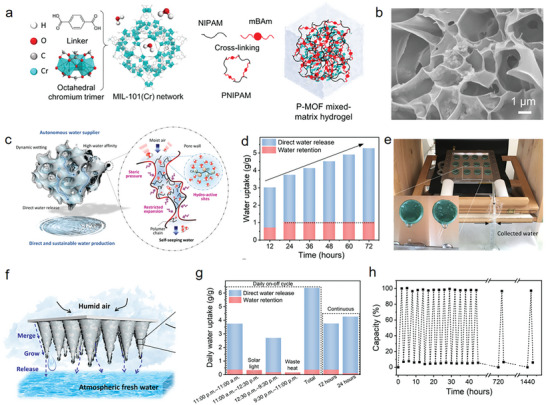
a) Schematic preparation and b) SEM image of the MOF‐polymer hydrogel. c) Diagram of the autonomous atmospheric water harvest system and d) ongoing water uptake performance of PC‐MOF. e) Optical image of atmospheric water harvest using PC‐MOF. f) Diagram image of the PCA‐MOF cone array. g,h) Daily water collection efficiency of the PCA‐MOF cone array. Reproduced with permission. Copyright 2020, AAAS.

MOFs are highly promising porous materials noted for their ability to capture water effectively even at relatively low humidity levels. This capability stems from their structural diversity and precisely tailored functionalization, which allows for the adjustment of pore size and hydrophilicity. These features collectively enhance the potential of MOFs for use in atmospheric water harvesting.^[^ ^69^, ^305^, ^306^, ^307^ ^]^ MOF‐polymer aerogels, created by embedding the water‐stable MIL‐101(Cr) nanoparticles into a poly(N‐isopropylacrylamide) (PNIPAM) framework, have been harnessed for autonomous atmospheric water absorption and retrieval as illustrated in **Figure** **21**a,b. Due to steric hindrance and the limited expansion of the PC‐MOF structure, the absorbed water condenses and gets expelled once sorption reaches saturation, as depicted in Figure 21c. The finely tuned PC‐MOF showcases an impressive water yield of 3.01 g g^−1^ after 12 h of sorption at 90% relative humidity (RH). Moreover, after a prolonged duration of 72 h dedicated to water collection at 90% RH, the material retains 1.02 g g^−1^, and the quantity of passively accumulated water surges to 4.25 g g^−1^, as shown in Figure 21d. An external field test of this technology was conducted using a prototype comprising an array of PC‐MOF aerogels. This setup underwent a 24 h exposure to the outdoors, facilitating concurrent water uptake and release, evidenced in Figure 21e. Furthermore, water was extracted by applying thermal activation to the system. This prototype demonstrated the capacity to harvest 4.20 g of water per gram of aerogel. In an innovation to boost efficiency, MIL‐101(Cr) particles were laden with gold (Au) nanoparticles before integration into the polymer (PCA‐MOF) to harness a photothermal effect. As demonstrated in Figure 21f, the PCA‐MOF aerogel, featuring a conical array geometry, exhibited exceptional water sorption capability—generating 6 g of fresh water per gram of sorbent at 90% RH—and maintained long‐term operational resilience, delivering water continuously over 1440 h (Figure 21g,h).^[^ ^308^ ^]^

### 4.4 Energy Storage

#### 4.4.1 Electrodes

The utilization of MOFs/COFs in electrode has been investigated recently.^[^ ^66^, ^309^, ^310^, ^311^ ^]^ Aggregation and restacking of MOF/COF powders can result in reduced permeability for electrolyte/reactant molecules and limited accessibility to internal active sites. Consequently, developing 3D porous hierarchical structures based on MOF/COF shows promise for practical energy storage applications, enabling higher mass loading, enhanced exposure of active sites, and accelerated mass/electron transport. In addition, COFs and MOFs show mostly very low electrical conductivities, if any. Incorporation of MOFs/COFs into 3D porous conducting structures such as graphene aerogels by aforementioned postsynthetic hybridization or in situ growth approaches is therefore an intriguing strategy for broadening their application as flexible electrodes in batteries and supercapacitors.^[^ ^312^ ^]^

**Figure 22:**
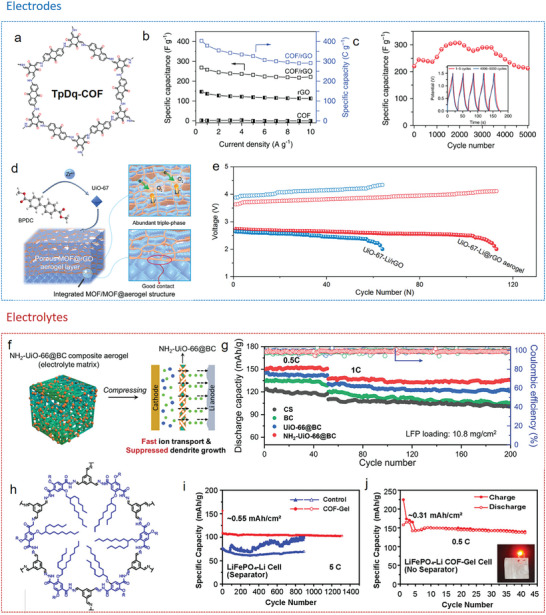
a) The structure of TpDq‐COF. b) The specific capacitances and capacities of COF/rGO aerogel under different current density. c) The cyclic stability of COF/rGO aerogel capacitor. Reproduced with permission.^[^ ^105^ ^]^ Copyright 2020, Springer Nature. d) The MOF/MOF@rGO aerogel architecture alongside an exposition of its structural benefits for Li‐O_2_ battery. e) Cycle performance of solid‐state Li‐O_2_ batteries based on UiO‐67‐Li/rGO and UiO‐67‐Li@rGO aerogel. Reproduced with permission.^[^ ^313^ ^]^ Copyright 2022, Wiley‐VCH. f) Diagrammatic representation of NH_2_‐UiO‐66@BC composite aerogel as a gel electrolyte matrix and its role in ion‐transfer in batteries. g) The cycle stability of various electrolytes of LiFePO_4_|Li cells. Reproduced with permission.^[^ ^152^ ^]^ Copyright 2022, Wiley‐VCH. h) The structure of COF used as gel electrolyte. i) Cycling performance of cells at 5 C. j) Cycling performance of cells at 0.5 C without separator skeleton. Reproduced with permission.^[^ ^314^ ^]^ Copyright 2022, Wiley‐VCH.

Flexible and lightweight supercapacitors have attracted much attention for wearable or portable electronic devices. Zhao et al. prepared a 2D Cu/Co‐tetrahydroxy‐1,4‐quinone (THQ)@CNTs@rGO aerogel cathode by introducing 2D conductive MOFs into 3D‐printed aerogels for lithium‐ion hybrid supercapacitor (LIHC). Leveraging the high capacity and “lubricant” properties of 2D Cu/Co‐THQ along with the extensive porosity of the 3D‐printed microlattice electrodes, the LIHC device exhibits robust cycle performance. It maintains a capacity retention of 89.43% after 4000 charge/discharge cycles at 2 A g^−1^, and achieves a Coulombic efficiency close to 100%.^[^ ^204^ ^]^ COFs has also been suggested as electrode materials for electrochemical capacitors. In our recent work, redox‐active anthraquinone based COF‐graphene aerogels were demonstrated as electrodes for supercapacitor without conducting additives and binders. Attributing to a synergistic effect where rGO enhances conductivity and the COF provides a high surface area and redox‐active sites, the device achieved a capacitance of 269 F g^−1^ at 0.5 A g^−1^, along with stable cycling over 5000 cycles (**Figure** **22**a–c). Additionally, the developed 3D network facilitated rapid charge transfer and ion diffusion to the redox‐active sites.^[^ ^105^ ^]^ Separately, Xu's group engineered a highly stable solid‐state Li‐O_2_ battery incorporating a Li‐ion‐conducting UiO‐67/rGO aerogel as the solid‐state cathode.^[^ ^313^ ^]^ The SSC is engineered by the in situ integration of MOF solid‐state electrolytes onto the porous rGO aerogel surface. This configuration ensures ample and continuous triple‐phase boundaries (TPBs) along with a robust solid‐solid contact interface. As a consequence, there is a synergistic facilitation of both Li ions and electrons transport, as well as a rapid diffusion of O_2_ molecules (Figure 22d). The solid‐state Li‐O_2_ battery, equipped with a UiO‐67‐Li@rGO aerogel, showcases improved cycling stability. It sustains up to 115 cycles before reaching the cut‐off voltage of 2 V, operating at a current density of 100 mA g^−1^ while consistently maintaining a capacity of 500 mAh g^−1^ (Figure 22e).

#### 4.4.2 Electrolyte Matrix

3D porous gel‐based electrolytes combine the advantages of high ionic conductivity with excellent interfacial characteristics, making them well‐suited as separators between two electrodes. Recently, 3D porous macrostructures crafted from MOFs and COFs are emerging as promising electrolyte matrices for battery applications.^[^ ^313^ ^]^

Fu et al. have showcased the NH_2_‐UiO‐66@BC composite aerogel electrolyte, which effectively curtails dendrite growth and promotes ion conduction.^[^ ^152^ ^]^ This NH_2_‐UiO‐66@BC aerogel electrolyte boasts a high ionic conductivity of ≈1 mS cm^−1^, a high lithium‐ion transference number of 0.82, and commendable electrochemical stability. Batteries equipped with the NH_2_‐UiO‐66@BC aerogel electrolyte demonstrated impressive long‐term cycling stability, maintaining a capacity of 134 mAh g^−1^ after 200 cycles at a 1 C rate. This performance outstrips that of cells utilizing UiO‐66@BC, bare carbon, and commercial separator electrolytes, evidenced by the higher capacities achieved at both 0.5 C and 1 C rates.^[^ ^152^ ^]^

Furthermore, a flexible COF gel electrolyte synthesized from 1,3,5‐trisformylbenzene and a hydrazide‐based monomer with a branched alkyl chain was developed for use in lithium‐ion batteries. Employing this electrolyte, the battery delivered an initial discharge capacity of 108.9 mAh g^−1^, and displayed remarkable capacity retention of 94.7% after more than 1300 cycles. Additionally, a pouch cell assembled with this COF gel electrolyte and without any additional separator framework successfully powered a light‐emitting diode (LED) lamp, showcasing its practical applicability.^[^ ^314^ ^]^

### 4.5 Catalysis

Aerogels have demonstrated enhanced catalytic activity when compared to their bulk counterparts.^[^ ^48^, ^315^ ^]^ In recent years, there has been progress in the creation of innovative aerogels based on MOFs and COFs for use in catalysis. This section delves into several case studies concerning electrocatalysis, photocatalysis, heterogeneous and nanozyme catalysis, with the aim of presenting an overview of the design principles behind MOF/COF‐based aerogel catalysts.

Electrocatalysis is crucial in the conversion of renewable energy, facilitating a variety of sustainable processes pivotal for the advancement of future technologies. The primary strategies to enhance the efficacy of electrocatalysts involve augmenting the accessibility of active sites and expediting mass transfer during catalysis. Aerogels offer an ideal structure that fulfills these key criteria for designing high‐performance electrocatalysts. Apart from the array of MOF‐derived nanomaterials that are integrated with carbon‐based aerogels, MOF/COF‐based aerogels have been recently engineered for electrocatalytic purposes in reactions such as the oxygen evolution reaction (OER) and the hydrogen evolution reaction (HER).^[^ ^316^ ^]^

In the CoNi‐BDC/carbon aerogel system, the enhanced mass transfer capabilities position it as a promising candidate for electrocatalytic materials. This system was specifically engineered as a free‐standing working electrode for OER. It demonstrated excellent OER catalytic efficiency, achieving an overpotential of 192 mV at 10 mA cm^−2^ and a Tafel slope of 39.4 mV dec^−1^. This performance can be attributed to the abundant accessible active sites and the superior mass transfer efficiency of the hierarchical CoNi‐MOF/aerogel structure.^[^ ^169^ ^]^

**Figure 23:**
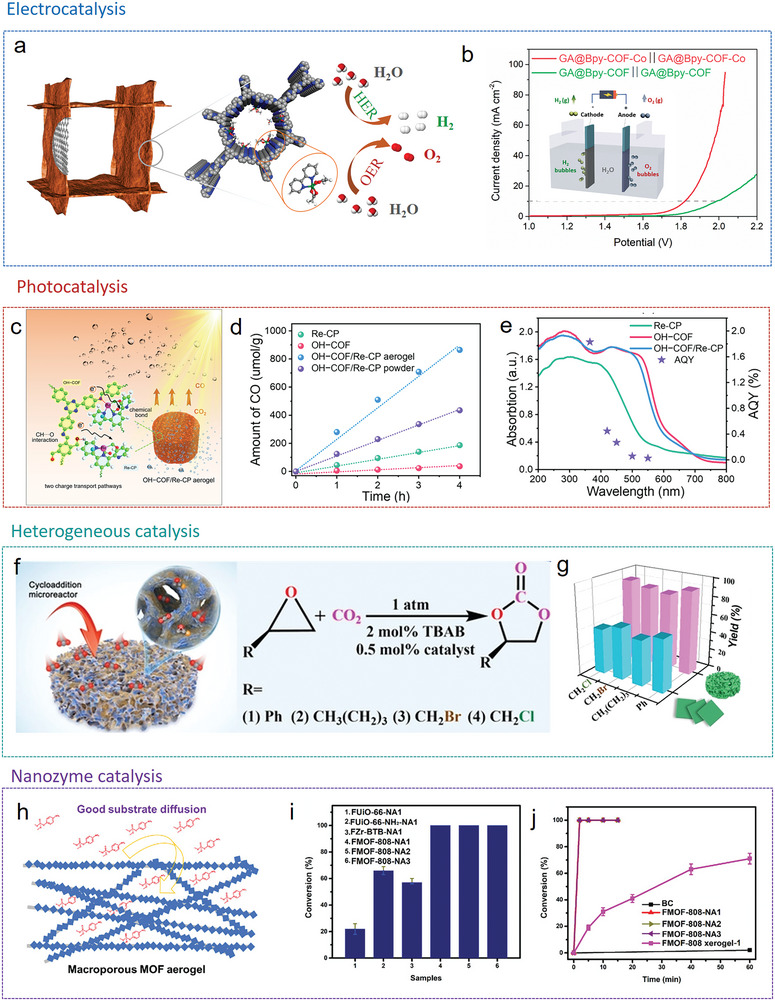
MOF/COF‐based aerogels for catalysis applications. Electrocatalysis: a) Schematic illustration of COF/GA. b) Polarization curve of GA@Bpy‐COF‐Co||GA@Bpy‐COF‐Co and the GA@Bpy‐COF||GA@Bpy‐COF. Inset: Schematic diagram of an electrolyzer for water splitting. Reproduced with permission.^[^ ^236^ ^]^ Copyright 2022, Royal Society of Chemistry. Photocatalysis: c) Schematic diagram of OH–COF/Re‐CP aerogel photocatalyst for gas–solid CO_2_ reduction. d) CO production performance and e) the AQY of various catalysts. Reproduced with permission.^[^ ^320^ ^]^ Copyright 2024, Cell Press. Heterogeneous catalysis: f) Schematic representation of the CuBDC/CMC microreactor catalyst for CO_2_ cycloaddition. g) Yield for cyclic carbonates. Reproduced with permission.^[^ ^323^ ^]^ Copyright 2021, Wiley‐VCH. h) Illustration of diffusion in macroporous MOF nanozyme aerogel. i) Conversion of DMNP using various fibrous Zr‐MOF nanozyme aerogel catalysts. j) Kinetics of DMNP conversion with different fibrous MOF‐808 nanozyme aerogel catalysts. Reproduced with permission.^[^ ^123^ ^]^ Copyright 2023, Wiley‐VCH.

In our recent study, we synthesized a cobalt‐enhanced COF/graphene aerogel electrocatalyst, designated GA@Bpy‐COF‐Co, by cultivating cobalt bipyridine‐COFs on amino‐functionalized graphene aerogel (GA), as illustrated in **Figure** **23**a. Thanks to a 3D conductive network and readily available catalytic sites, this aerogel exhibited outstanding OER and HER activity, achieving overpotentials of only 300 mV for OER and 275 mV for HER, both measured at a current density of 10 mA cm⁻^2^. We developed an electrolyzer for water splitting by integrating GA@Bpy‐COF‐Co as both the anode and cathode, as depicted in Figure 23b. The device exhibited a cell voltage of 1.82 V at 10 mA cm⁻^2^ and successfully produced hydrogen and oxygen at rates of 1.14 and 0.58 µL s^−1^, respectively, under standard atmospheric conditions.^[^ ^236^ ^]^

Unlike powders or granules, the interconnected and open framework of aerogels enhances the mass transfer and charge transport, resulting in better reactant accessibility, advantageous for photocatalysis.^[^ ^317^, ^318^, ^319^ ^]^ Recent research designed a COF‐based Z‐scheme heterostructure aerogel, composed of hydroxy‐functionalized COFs (OH–COF) and poly(terpyridine)metal complex (Re‐CP).^[^ ^320^ ^]^ The integration of multiple interactions at the interface of the heterojunction boosts charge transfer kinetics and photocatalytic CO_2_‐to‐CO activity in gas‐solid CO_2_ reduction without using sacrificial agents or photosensitizers (Figure 23c). The CO production rate of OH–COF/Re‐CP reaches 216.2 µmol g^−1^ h^−1^, significantly surpassing that of the OH–COF/Re‐CP powder at 108.7 µmol g^−1^ h^−1^ (Figure 23d). Moreover, the apparent quantum yield (AQY) values of OH–COF/Re‐CP aerogel are 1.83% and 0.45% at 365 and 420 nm (Figure 23e), respectively. This work presents a promising aerogel photocatalyst with multiple interactions, offering a novel strategy to enhance photo‐induced dynamics at heterointerfaces, guiding the design of efficient COF‐based aerogel photocatalysts.

Additionally, MOF‐based aerogels also show great potential in the field of heterogeneous catalysis.^[^ ^169^, ^321^, ^322^ ^]^ For example, a hierarchically structured Cu‐BDC/CMC aerogel was furthermore utilized as catalyst for the cycloaddition of CO_2_ with epoxides to produce cyclic carbonates (Figure 23f). The lamellar Cu‐BDC film on the aerogel provided a multitude of accessible active sites, enabling effective adsorption of reactant molecules for catalysis. As a result, the yield of phenyl cyclic carbonate reached up to 91.5% at 70 °C over 18 h, which is 1.6 times higher than the yield obtained using Cu‐BDC nanosheet catalysts under identical conditions (Figure 23g).

Chemical warfare agents (CWAs) remain a serious threat to both human safety and national security. The catalytic neutralization of these hazardous substances has been successfully realized through the application of MOF‐based aerogels. These MOF‐based aerogels provide a promising platform for efficiently detoxifying toxic warfare agents^[^ ^142^, ^323^, ^324^, ^325^ ^]^ Farha's group proposed a fibrous MOF‐808 aerogel composites with high MOF content (90 wt%), high surface areas (1520 m^2^ g^−1^), and impressive stability.^[^ ^123^ ^]^ The integration of a macroporous cellulose nanofiber aerogel matrix with a microporous Zr‐MOF nanozyme significantly facilitates the accessibility to the active sites for catalysis, as depicted in Figure 23h. These fibrous MOF nanozyme aerogel composites, referred to as FMOF‐808‐NAs, have been rigorously evaluated for their ability to decompose the organophosphorus nerve agent simulant, dimethyl‐4‐nitrophenyl phosphate (DMNP), in a 0.4 m N‐ethylmorpholine solution. Relative to UiO‐66, UiO‐66‐NH_2_, and Zr‐BTB MOFs, shown in Figure 23i, the FMOF‐808‐NAs demonstrate superior conversion rates. Remarkably, the FMOF‐808‐NAs achieved complete DMNP conversion within just 2 min. On the other hand, the MOF‐808‐xerogel required a half‐life of 30 min for DMNP hydrolysis, as illustrated in Figure 23j, confirming that agent diffusion is significantly improved compared with the less microporous structure found in xerogel samples.

## 5 Summary and Outlook

**Figure 24:**
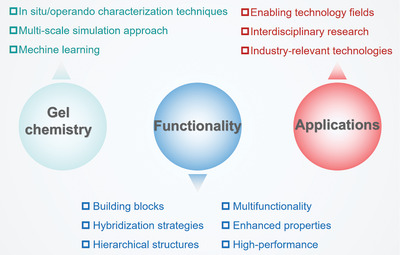
Challenges and future trends of MOF/COF‐based aerogels.

Over the last ten years, the field of MOF/COF‐based aerogels has flourished, with significant advancements propelled by their distinct physicochemical attributes and hierarchically porous architectures. A variety of synthesis strategies and practical applications have been explored, substantially fortifying the foundational understanding of these materials while expanding the arsenal of potential uses. This review not only presents the common synthesis routes and gelation mechanisms of pure MOF and COF aerogels, but it also shines a light on hybridization techniques involving other functional components, such as (bio)polymers, graphene, and MXene. It offers a comprehensive survey of the advocated applications, aiming to serve as a guide for scientists across various disciplines who are eager to enter and contribute to this nascent yet rapidly evolving area of study. Despite the impressive capabilities demonstrated by numerous advanced MOF/COF aerogels and their hybrid counterparts in a diversity of applications, there remain critical challenges that warrant further investigation within this dynamic field in the coming years (**Figure** **24**).

A key aspect of gel chemistry is that even minor modifications in the interacting components can lead to different structures. Specifically for pure MOF/COF aerogels, the intricate balance between the kinetics and thermodynamics of their gelation process is dictated by the variety of intermolecular interactions within their 3D network. Gaining a thorough insight into these interactions is vital for the development of MOF/COF aerogels with customizable properties. To achieve this, there is a pressing need for the implementation of in situ/operando characterization techniques and molecular dynamic simulation method. These techniques are crucial for understanding the micro/macroscopic behavior of MOF/COF gels, alongside the fundamental structure–property correlations.

Real‐time monitoring of structural changes of MOFs and COFs during sol‐gel transformation process can be achieved through various techniques. In situ X‐ray diffraction (XRD) and small‐angle X‐ray scattering (SAXS) can be used to monitor crystallinity and phase transitions, as well as observe changes in pore structure and network formation. Spectroscopic methods (e.g., FTIR, Raman) can track chemical bonding and functional group changes, while electron microscopy helps visualize morphological evolution.^[^ ^326^ ^]^ These techniques contribute to the understanding of reaction kinetics and mechanisms, identification of intermediate species and phases, and correlation between synthesis conditions and final material properties. Complementing experimental data with theoretical insights, molecular dynamic simulation methods provide predictions of material behavior at atomic and molecular levels, enhance understanding of interactions between building blocks of MOFs/COFs, and allow for the virtual exploration of various synthesis conditions.

In the future, machine learning‐assisted analysis of in situ data will enhance our ability to interpret complex experimental results more efficiently and accurately.^[^ ^327^ ^]^ High‐throughput screening of MOF and COF‐aerogel compositions will accelerate the discovery and optimization of new materials with tailored properties for specific applications.^[^ ^328^, ^329^ ^]^ Additionally, the development of multi‐scale simulation approaches will bridge the gap between atomic‐level simulations and macroscopic material properties, providing a more comprehensive understanding of material behavior.^[^ ^330^ ^]^ These advancements will collectively contribute to more rapid and efficient materials design and characterization processes of MOF and COF‐aerogels.

To preserve the intricate 3D network structures of MOF/COF gels during the transition from liquid to gas phase, supercritical drying is commonly used. However, this technique is time‐intensive, technically challenging, and restricted to batch processing. Therefore, it is essential to devote more resources to enhancing alternative drying methods, such as ambient pressure drying, to improve their practicality. Typically, the mechanical strength of pure MOF/COF aerogels is significantly weak, largely due to their crystalline frameworks which are inherently more brittle compared to the amorphous structure found in traditional aerogel materials. Strengthening MOFs/COFs by hybridizing them with mechanically stable components is an effective strategy to mitigate this fragility, producing MOF/COF‐based aerogels with enhanced robustness. Moreover, the introduction of a hierarchical network structure might also enhance the aerogels' stability. Additionally, assembling 1D or 2D MOF nanostructures into a 3D network through templating methods emerges as a promising approach for creating resilient and elastic aerogels.

The exploration of the underlying 3D self‐assembly chemistry between MOFs/COFs and other low‐dimensional building blocks requires further investigation to foster the development of additional synthetic approaches and novel MOF/COF hybrid aerogels. The detailed microstructure within these hybrid aerogels, particularly the distribution of MOF or COF nanocrystals and their interfaces, remains partially obscure and necessitates more in‐depth research to truly understand the connection between structure and properties. Introducing a wider array of 0D, 1D, and 2D MOFs, as well as 2D COFs, into aerogels holds the potential to broaden their functionalities and applications substantially.

Currently, aerogels derived from MOF/COF nanocrystals are not available at an industrial scale, and the field remains primarily in the realm of basic research. A greater emphasis on the translation of laboratory‐scale processes to industry‐relevant technologies is imperative to reduce manufacturing costs and facilitate the commercial adoption of these materials.

To address these challenges and capitalize on future trends, interdisciplinary research collaboration will be crucial. This includes combining expertise from materials science, chemistry, engineering, and application‐specific fields. Additionally, advances in characterization techniques and computational modeling will play a significant role in understanding and optimizing the properties of MOF and COF‐based aerogels. As research progresses, we can expect to see more tailored and high‐performance MOF and COF‐based aerogels that address specific technological needs across various industries, potentially revolutionizing fields such as energy, environment, healthcare, and advanced manufacturing.
